# Household Secondary Attack Rates of SARS-CoV-2 by Variant and Vaccination Status: An Updated Systematic Review and Meta-analysis

**DOI:** 10.1001/jamanetworkopen.2022.9317

**Published:** 2022-04-28

**Authors:** Zachary J. Madewell, Yang Yang, Ira M. Longini, M. Elizabeth Halloran, Natalie E. Dean

**Affiliations:** 1 Department of Biostatistics, University of Florida, Gainesville; 2 Fred Hutchinson Cancer Research Center, Seattle, Washington; 3 Department of Biostatistics, University of Washington, Seattle; 4 Department of Biostatistics and Bioinformatics, Emory University, Atlanta, Georgia

## Abstract

**Importance:**

An overall household secondary attack rate (SAR) of 18.9% (95% CI, 16.2%-22.0%) through June 17, 2021 was previously reported for SARS-CoV-2. Emerging variants of concern and increased vaccination have affected transmission rates.

**Objective:**

To evaluate how reported household SARs changed over time and whether SARs varied by viral variant and index case and contact vaccination status.

**Data Sources:**

PubMed and medRxiv from June 18, 2021, through March 8, 2022, and reference lists of eligible articles. Preprints were included.

**Study Selection:**

Articles with original data reporting the number of infected and total number of household contacts. Search terms included *SARS-CoV-2*, *COVID-19*, *variant*, *vaccination*, *secondary attack rate*, *secondary infection rate*, *household*, *index case, family contacts*, *close contacts,* and *family transmission.*

**Data Extraction and Synthesis:**

The Preferred Reporting Items for Systematic Reviews and Meta-Analyses reporting guideline was followed. Meta-analyses used generalized linear mixed models to obtain SAR estimates and 95% CIs.

**Main Outcomes and Measures:**

SAR stratified by covariates according to variant, index case and contact vaccination status, and index case identification period. SARs were used to estimate vaccine effectiveness on the basis of the transmission probability for susceptibility to infection (*VE_S,p_*), infectiousness given infection (*VE_I,p_*), and total vaccine effectiveness (*VE_T,p_*).

**Results:**

Household SARs were higher for 33 studies with midpoints in 2021 to 2022 (37.3%; 95% CI, 32.7% to 42.1%) compared with 63 studies with midpoints through April 2020 (15.5%; 95% CI, 13.2% to 18.2%). Household SARs were 42.7% (95% CI, 35.4% to 50.4%) for Omicron (7 studies), 36.4% (95% CI, 33.4% to 39.5%) for Alpha (11 studies), 29.7% (95% CI, 23.0% to 37.3%) for Delta (16 studies), and 22.5% (95% CI, 18.6% to 26.8%) for Beta (3 studies). For full vaccination, *VE_S,p_* was 78.6% (95% CI, 76.0% to 80.9%) for Alpha, 56.4% (95% CI, 54.6% to 58.1%) for Delta, and 18.1% (95% CI, −18.3% to 43.3%) for Omicron; *VE_I,p_* was 75.3% (95% CI, 69.9% to 79.8%) for Alpha, 21.9% (95% CI, 11.0% to 31.5%) for Delta, and 18.2% (95% CI, 0.6% to 32.6%) for Omicron; and *VE_T,p_* was 94.7% (95% CI, 93.3% to 95.8%) for Alpha, 64.4% (95% CI, 58.0% to 69.8%) for Delta, and 35.8% (95% CI, 13.0% to 52.6%) for Omicron.

**Conclusions and Relevance:**

These results suggest that emerging SARS-CoV-2 variants of concern have increased transmissibility. Full vaccination was associated with reductions in susceptibility and infectiousness, but more so for Alpha than Delta and Omicron. The changes in estimated vaccine effectiveness underscore the challenges of developing effective vaccines concomitant with viral evolution.

## Key Points

**Question:**

Are viral variants of concern and increased vaccination associated with SARS-CoV-2 household transmission rates?

**Findings:**

In this systemic review and meta-analysis of 135 studies with more than 1.3 million participants in 36 countries, household secondary attack rates increased over time and were higher for Omicron (42.7%), Alpha (36.4%), and Delta (29.7%) variants than previously reported estimates (18.9%). Full vaccination reduced susceptibility and infectiousness, but more so for Alpha than Delta and Omicron.

**Meaning:**

These findings suggest vaccination for SARS-CoV-2 transcends protection of the individual by conferring indirect protection to other household members, but the degree of protection is seemingly lower for emerging variants.

## Teaser

This systematic reviewe and meta-analysis evaluates household secondary attack rates of SARS-CoV-2 by variant and vaccination status.

## Introduction

A previously published SARS-CoV-2 household transmission meta-analysis^1^ through June 17, 2021, reported an overall secondary attack rate (SAR) of 18.9% (95% CI, 16.2%-22.0%) for SARS CoV-2. Although COVID-19 vaccines are more widely available to protect household contacts, emerging variants such as Omicron (B.1.1.529) are even more transmissible and are known to evade immunity induced by existing vaccines or natural infections with the original wild type.^2^ The net impact of emerging variants on household transmission in vaccinated and unvaccinated households is of interest. More importantly, household SAR studies can also yield estimates of vaccine effectiveness (VE), that is, the association between vaccination and susceptibility to infection, infectiousness given infection, and the total direct and indirect benefits associated with vaccinated individuals in vaccinated populations.^3,4^ In this meta-analysis, we aggregate household contact tracing studies to evaluate SARs for variants and by index case and contact vaccination status.

## Methods

This study followed the Preferred Reporting Items for Systematic Reviews and Meta-analyses (PRISMA) reporting guideline using the same definitions and eligibility criteria as our original study.^5^ We estimated household transmissibility of SARS-CoV-2 by calculating the SAR or the number of new infections among contacts after exposure to an index case divided by the total number of household contacts. Our last review identified studies published through June 17, 2021.^1^ Herein, we searched PubMed, medRxiv, and reference lists of eligible studies between June 18, 2021, and March 8, 2022, with no restrictions on language, study design, or place of publication. Search terms were *SARS-CoV-2*, *COVID-19*, *severe acute respiratory syndrome*, *SARS*, *SARS-CoV*, *coronavirus*, *variant*, *vaccination*, *immunization*, *secondary attack rate*, *secondary infection rate*, *household*, *family contacts*, *close contacts*, *index case*, *contact transmission*, *contact attack rate*, and *family transmission* (eTable 1 in the Supplement). Preprints were included. Citations were managed in EndNote version 20 (Thomson Reuters).

Articles with original data that reported at least 2 of the following factors were included: number of infected household contacts, total number of household contacts, and household SARs. Studies that reported only infection prevalence, analyzed populations that overlapped with another included study, and tested contacts using antibody tests only or using antibody tests and another test but did not disaggregate SARs by test were excluded. We first screened studies by titles and abstracts to identify potential studies for inclusion. We then evaluated full-text articles and selected those that met the inclusion criteria.

For this study, 1 reviewer (Z.J.M.) extracted the following information: first author, location, index case identification period, number of index cases, index case symptom status, household/family contact type, test used to diagnose contacts, universal/symptomatic testing, number of tests per contact, and follow-up duration. That reviewer also extracted the number of infected household contacts and total number of household contacts and, whenever possible, disaggregated by covariates including viral variant, index case vaccination status, household contact vaccination status, and vaccine type.

### Evaluation of Study Quality and Risk of Bias

To assess study quality and risk of bias, we used the same modified version of the Newcastle-Ottawa quality assessment scale used by Fung et al.^6^ Studies received up to 9 points according to participant selection (4 points), study comparability (1 point), and outcome of interest (4 points). Studies were classified as having high (≤3 points), moderate (4-6 points), and low (≥7 points) risk of bias. When at least 10 studies were available, we also used funnel plots and Begg and Mazumdar rank correlation to evaluate publication bias, with significance set at *P* < .10.^7^ If we detected publication bias, we used the trim-and-fill method for adjustment, which consists of imputing missing effect sizes to achieve symmetry.^8^

### Statistical Analysis

To examine temporal patterns, we assessed household SARs by index case identification period midpoint. We restricted this analysis to laboratory-confirmed infections and SARs from unvaccinated index cases to unvaccinated contacts to observe how transmission patterns changed by time independently of vaccination. We then evaluated household SARs by variants that were reported in 2 or more studies regardless of vaccination status and restricted to SARs from unvaccinated index cases to unvaccinated contacts for comparison with SAR estimates from our original analyses of the predominantly wild-type variant. SAR statistical analyses by variant were as previously described.^1^

We evaluated SARs by index case and household contact vaccination status (unvaccinated, partially vaccinated, fully vaccinated, booster vaccinated, and all) by variant and overall across variants. The resultant SARs were used to estimate vaccine effectiveness for reducing susceptibility (*VE_S,p_*) and infectiousness (*VE_I,p_*) according to the transmission probability *p*.^3,4^ We calculated *VE_S,p_* from the studies included using *VE_S,p_* = 1 − *SAR*_01_ / *SAR*_00_ or *VE_S,p_* = 1 − *SAR*_11_ / *SAR*_10_, and *VE_1,p_* from the studies included using *VE_I,p_* = 1 − *SAR*_10_ / *SAR*_00_ or *VE_I,p_* = 1 − *SAR*_11_ / *SAR*_01_, where *SAR_ij_* represent the SAR associated with vaccine status *i* (1 = vaccinated, 0 = unvaccinated) for the index case and *j* for the household contact. Total estimated vaccine effectiveness is defined as *VE_T,p_* = 1 − (1 − *VE_S,p_*) × (1 − *VE_I,p_*).

For comparing vaccination subgroups, we conducted pairwise analyses using only studies in which SARs were reported from both relevant subgroups. For VE measures, we used generalized linear mixed-effects models to obtain SAR logits and corresponding sampling variances, which were back-transformed to obtain VE summary estimates and 95% CIs. Furthermore, we used generalized linear mixed-effects models to compare SARs by vaccine type for contact vaccination status with study treated as a random effect and vaccine type as a fixed effect moderator. Heterogeneity was measured using the *I^2^* statistic, with thresholds of 25%, 50%, and 75% indicating low, moderate, and high heterogeneity, respectively. All analyses were performed using the metafor package in R statistical software version 4.1.2 (R Project).^9,10^ Statistical significance was set at a 2-tailed *P* ≤ .05.

## Results

**Figure 1.**
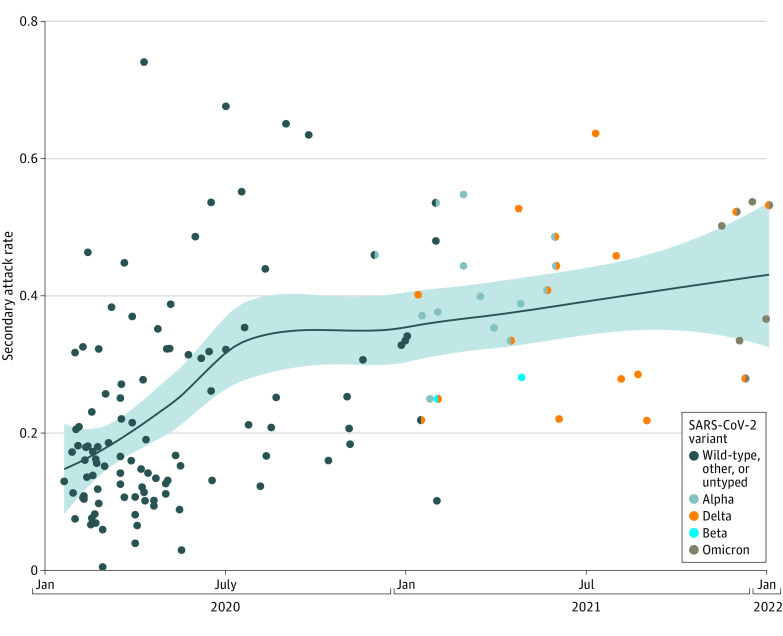
Household Secondary Attack Rates Over Time, by Study Midpoint, in 135 Studies of Unvaccinated Index Cases and Unvaccinated Contacts Data were restricted to laboratory-confirmed results only. The blue line is a loess smoothing line, and shaded bands are 95% CIs. Bicolored points represent studies with 2 predominant variants.

We identified 2097 records (1791 from PubMed, 306 from medRxiv, and 2 from reference lists of eligible articles) published between June 18, 2021, and March 8, 2022 (eFigure 1 in the Supplement). Fifty-eight new studies^11,12,13,14,15,16,17,18,19,20,21,22,23,24,25,26,27,28,29,30,31,32,33,34,35,36,37,38,39,40,41,42,43,44,45,46,47,48,49,50,51,52,53,54,55,56,57,58,59,60,61,62,63,64,65,66^ (eTable 2 in the Supplement) were combined with 77 studies from our previous review^1^ (see eTable 3 in the Supplement for references included from our previous review), with Figure 1 showing household SAR by study period, resulting in 135 total studies representing 1 375 806 contacts from 36 countries. Four of the new studies^47,48,49,58^ were preprints in our previous review that were subsequently published.

To assess trends in SAR over time distinct from trends in vaccination, we first restricted attention to unvaccinated index with unvaccinated household contacts. Despite large heterogeneity in SAR estimates over time, particularly during the early stages of the pandemic, an increasing trend is visible in Figure 1. The overall household SAR for 33 studies with midpoints in 2021 or 2022 was 37.3% (95% CI, 32.7%-42.1%), whereas the overall household SAR for 63 studies with midpoints through April 2020 was 15.5% (95% CI, 13.2%-18.2%) (see eTable 3 in the Supplement for references). Begg and Mazumdar rank correlation for publication bias was significant for studies in 2021 to 2022 (*P* < .001; Kendall τ, 0.664) but not studies through April 2020 (eFigure 2 in the Supplement).

**Figure 2.**
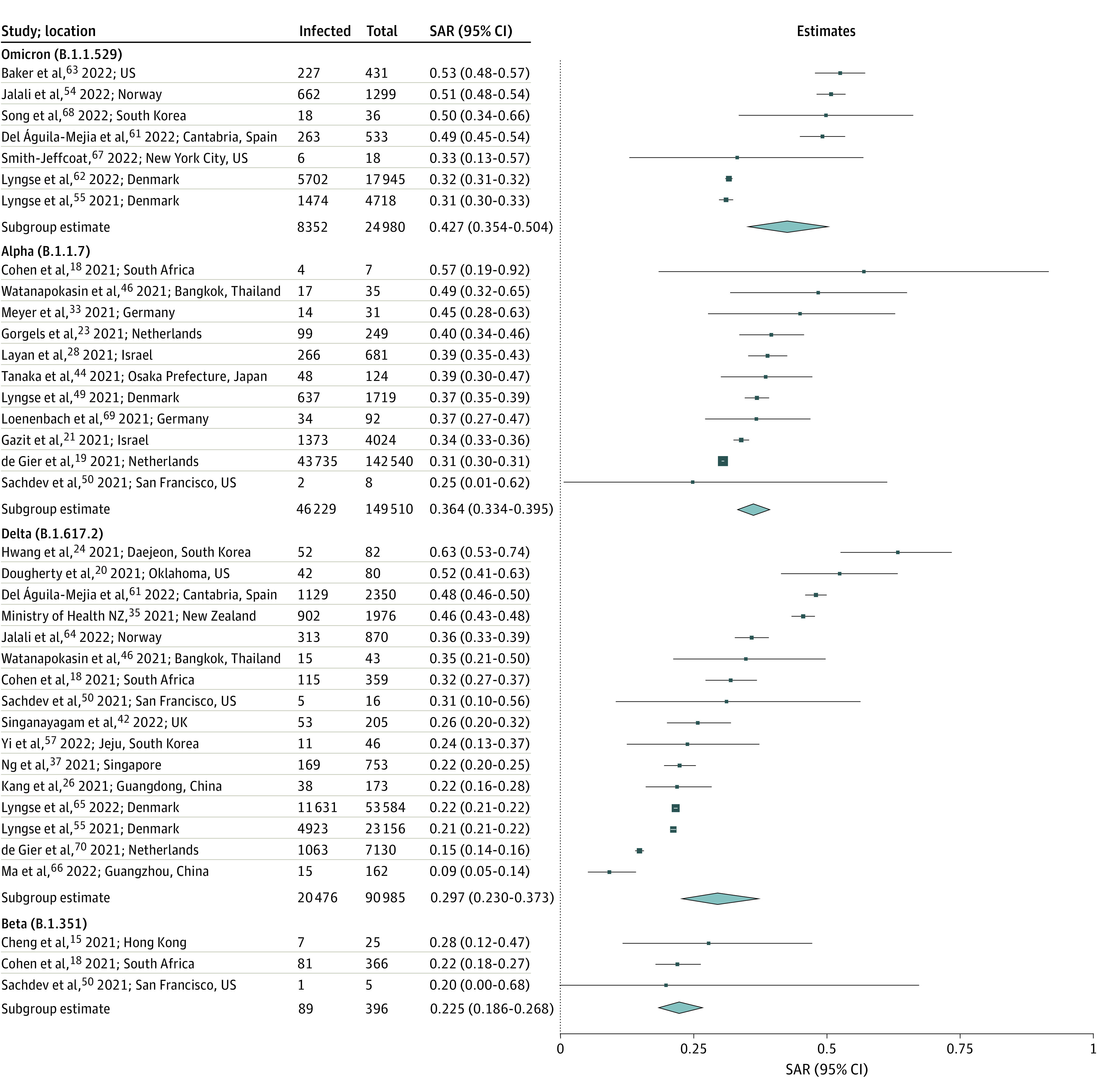
Household Secondary Attack Rates (SARs) for Omicron (B.1.1.529), Alpha (B.1.1.7), Delta (B.1.617.2), and Beta (B.1.351) Variants SARs included all index cases and contacts regardless of vaccination status. Point sizes (squares) are an inverse function of the precision of the estimates, and bars correspond to 95% CIs. Diamonds represent summary SAR estimates with corresponding 95% CIs. Heterogeneity indexes were as follows: Omicron (*I^2^* = 98.2%), Alpha (*I^2^* = 59.6%), Delta (*I^2^* = 99.1%), and Beta (*I^2^* = 2.6%).

Next, we estimated overall household SARs regardless of index case or contact vaccination status by viral variant. This reflects new variants and changing vaccination coverage. From highest to lowest, the overall household SARs were 42.7% (95% CI, 35.4%-50.4%) for Omicron (7 studies^55,61,62,63,64,67,68^), 36.4% (95% CI, 33.4%-39.5%) for Alpha (11 studies^18,19,21,23,28,33,44,46,49,50,69^), 29.7% (95% CI, 23.0%-37.3%) for Delta (16 studies^18,20,24,26,35,37,42,46,50,55,57,61,64,65,66,70^), and 22.5% (95% CI, 18.6%-26.8%) for Beta (3 studies^15,18,50^) (Figure 2). High heterogeneity was found among studies for Omicron (*I^2^* = 98.2%; *P* < .001) and Delta (*I^2^* = 99.1%; *P* < .001), moderate for Alpha (*I^2^* = 59.6%; *P* < .001), and low for Beta (*I^2^* = 2.6%; *P* = .79). Moderate asymmetry was observed in the funnel plot for studies of Alpha, which was significant from Begg and Mazumdar rank correlation (*P* = .09; Kendall τ, 0.418) (eFigure 3 in the Supplement). We therefore applied the trim-and-fill method, which yielded a mean SAR of 36.1% (95% CI, 33.2%-39.0%) for Alpha. We compared the variant-specific SARs regardless of vaccination status to variant-specific SARs estimated to unvaccinated household contacts only. The mean SAR changed most for Delta (37.0%; 95% CI, 29.7%-44.8%) (12 studies^18,20,24,37,42,46,55,57,61,64,65,70^) among the variants examined (eFigure 4 in the Supplement).

**Figure 3.**
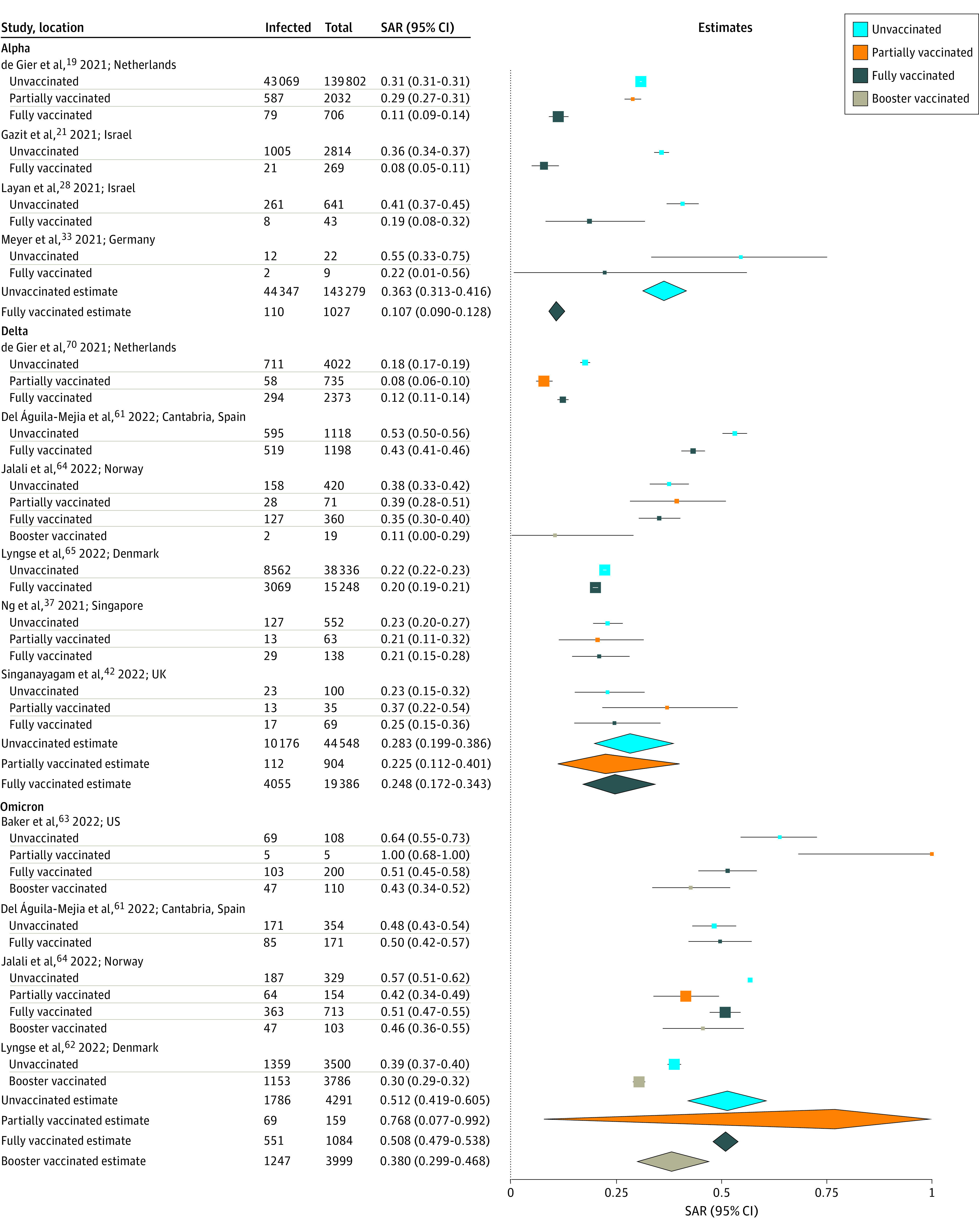
Household Secondary Attack Rates (SARs) by Index Case Vaccination Status All contacts are included regardless of vaccination status. For Harris et al,^48^ most of the vaccinated index cases (93%) had received only the first dose of vaccine and SARs were not disaggregated by dose. Point sizes (squares) are an inverse function of the precision of the estimates, and bars correspond to 95% CIs. Diamonds represent summary SAR estimates with corresponding 95% CIs. Heterogeneity indexes are as follows: Alpha (unvaccinated: *I^2^* = 94.6%; fully vaccinated: *I^2^* = 52.7%), Delta (unvaccinated: *I^2^* = 99.1%; partially vaccinated: *I^2^* = 91.7%; fully vaccinated: *I^2^* = 98.6%), and Omicron (unvaccinated: *I^2^* = 93.5%; partially vaccinated: *I^2^* = 70.5%; fully vaccinated: *I^2^* = 0.4%; booster vaccinated: *I^2^* = 78.5%).

To determine whether there are differences in infectiousness depending on the vaccination status of the index case, we conducted pairwise analyses of studies in which SARs were reported from both subgroups (eg, fully vaccinated vs unvaccinated index cases). Twelve studies^19,21,28,33,37,42,48,61,63,64,65,70^ reported SARs by index case vaccination status to all household contacts regardless of vaccination status (eFigure 5 in the Supplement), 11 of which were at low risk of bias, and 1 of which was moderate (eTable 4 in the Supplement). Estimated mean SAR for all variants combined was not significantly different from fully vaccinated index cases (22.8%; 95% CI, 15.3%-32.7%) than from unvaccinated (35.5%; 95% CI, 27.3%-44.6%; *P* = .05) (11 study pairs^19,21,28,33,37,42,61,63,64,65,70^), from partially vaccinated (26.2%; 95% CI, 11.5%-49.2%) than from unvaccinated (28.0%; 95% CI, 17.3%-42.0%; *P* = .12) (7 study pairs^19,37,42,48,63,64,70^), and from fully vaccinated (24.9%; 95% CI, 14.6%-39.2%) than from partially vaccinated (31.7%; 95% CI, 15.0%-55.0%; *P* = .62) (6 study pairs^19,37,42,63,64,70^) to all contacts regardless of vaccination status (eTable 5 in the Supplement). No significant publication bias was observed for studies of fully vaccinated or unvaccinated index cases (eFigure 6 in the Supplement). For 4 studies^19,21,28,33^ of Alpha variant, estimated mean SAR was significantly higher from unvaccinated index cases (36.3%; 95% CI, 31.3%-41.6%) than from fully vaccinated index cases (10.7%; 95% CI, 9.0%-12.8%; *P* < .001) (Figure 3). We found no significant difference in SARs by index case vaccination status for Delta and Omicron variants, but few studies were included in those subanalyses (Figure 3). Restricting to unvaccinated household contacts (eFigure 7 in the Supplement), estimated mean SAR was also significantly higher from unvaccinated index cases (30.9%, 95% CI, 23.9%-38.8%) than from fully vaccinated index cases (12.0%, 95% CI, 10.0%-14.2%; *P* < .001) in 4 paired studies,^19,21,42,56^ 2 of which^19,21^ included Alpha and 2 of which^42,70^ included Delta variants.

**Figure 4.**
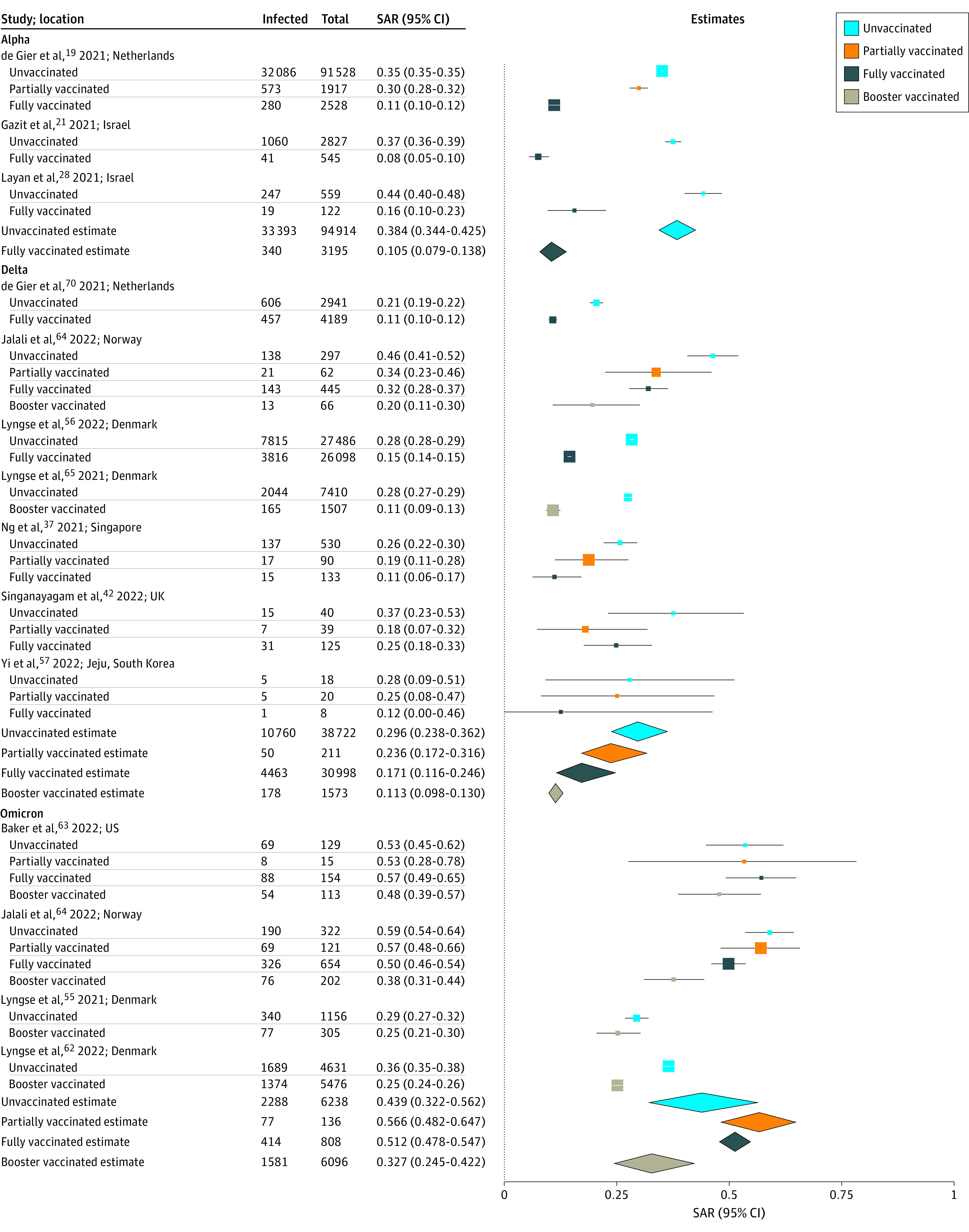
Household Secondary Attack Rates (SARs) by Contact Vaccination Status All index cases are included regardless of vaccination status. Point sizes (squares) are an inverse function of the precision of the estimates, and bars correspond to 95% CIs. Diamonds represent summary SAR estimates with corresponding 95% CIs. Heterogeneity indexes are as follows: Alpha (unvaccinated: *I^2^* = 92.5%; fully vaccinated: *I^2^* = 69.5%), Delta (unvaccinated: *I^2^* = 95.4%; partially vaccinated: *I^2^* = 30.4%; fully vaccinated: *I^2^* = 96.8%; booster vaccinated: *I^2^* = 76.5%), and Omicron (unvaccinated: *I^2^* = 97.1%; partially vaccinated: *I^2^* = 0.3%; fully vaccinated: *I^2^* = 60.1%; booster vaccinated: *I^2^* = 92.5%).

We then evaluated whether there were differences in susceptibility to SARS-CoV-2 infection depending on household contact vaccination status, again restricting to pairwise comparisons of studies reporting SARs for both relevant subgroups. eFigure 8 in the Supplement summarizes 12 studies^19,21,28,32,37,42,50,57,63,64,65,70^ reporting household SARs by contact vaccination status regardless of index case vaccination status, 10 of which were at low risk of bias and 2 of which were moderate. In 12 study pairs,^19,21,28,32,37,42,50,57,63,64,65,70^ estimated mean SAR for all variants combined was significantly higher for unvaccinated contacts (36.5%; 95% CI, 30.5%-43.0%) than for fully vaccinated contacts (18.8%; 95% CI, 12.6%-27.1%; *P* < .001). In 8 study pairs^32,37,42,50,57,63,64,70^ reporting SAR to partially vaccinated contacts (27.8%; 95% CI, 20.0%-37.1%), estimated mean SAR was not significantly different than to unvaccinated contacts (39.6%; 95% CI, 32.3%-47.4%; *P* = .08) or to fully vaccinated contacts (23.9%; 95% CI, 14.7%-36.4%; *P* = .66) (eTable 6 in the Supplement). Begg test did not show significant evidence of publication bias for studies of fully vaccinated or unvaccinated contact status (eFigure 9 in the Supplement). SARs were consistent when restricting to unvaccinated index cases only (eFigure 10 in the Supplement). When examining SARs by viral variant, estimated mean SARs were significantly higher for unvaccinated contacts for Alpha (38.4%; 95% CI, 34.4%-42.5%; *P* < .001) (3 studies^19,21,28^) and Delta (30.1%; 95% CI, 23.2%-38.1%; *P* = .01) (6 studies^37,42,57,64,65,70^) than for fully vaccinated contacts (Alpha: 10.5%; 95% CI, 7.9%-13.8%; Delta: 17.1%; 95% CI, 11.6%-24.6%) (Figure 4). For 2 studies^55,64^ of Delta, estimated mean SAR was also significantly higher for unvaccinated contacts (36.1%; 95% CI, 24.2%-50.0%) than for booster-vaccinated contacts (11.3%; 95% CI, 9.8%-13.0%; *P* < .001). For 4 studies^55,62,63,64^ of Omicron, SARs were not significantly different for unvaccinated contacts (43.9%; 95% CI, 32.2%-56.2%) than booster-vaccinated contacts (32.7%; 95% CI, 24.5%-42.2%; *P* = .16). SARs were generally lower for fully vaccinated contacts regardless of index case vaccination status (eFigure 11 in the Supplement).

We also examined SARs by vaccine type and contact vaccination status regardless of index case vaccination status where reported. In 4 study pairs,^19,32,50,65^ estimated mean SARs for household contacts fully vaccinated with Ad26.COV2.S (Janssen) (1 dose) (34.2%, 95% CI, 14.4%-61.5%) or BNT162b2 (Pfizer-BioNTech) (2 doses) (15.2%, 95% CI, 14.6%-16.0%) were significantly higher than those for contacts fully vaccinated with mRNA-1273 (Moderna) (2 doses) (9.5%, 95% CI, 8.6%-10.6%; *P* < .001) (eTable 7 in the Supplement). In 2 study pairs,^32,70^ estimated mean SAR was higher for contacts partially vaccinated with ChAdOx1 (AstraZeneca) (29.5%; 95% CI, 24.0%-35.7%) than contacts partially vaccinated with mRNA-1273 (17.5%; 95% CI, 13.7%-22.3%; *P* = .008). There was no significant difference in SAR for contacts fully vaccinated with ChAdOx1 and BNT162b2, Ad26.COV2.S, or RNA-1273; or for contacts partially vaccinated with BNT162b2 and RNA-1273 or ChAdOx1.

We also estimated vaccine effectiveness based on the SARs without considering vaccine type (Table). For full vaccination, estimated *VE_S,p_* (vaccine effectiveness for susceptibility) was 78.6% (95% CI, 76.0% to 80.9%) for Alpha, 56.4% (95% CI, 54.6% to 58.1%) for Delta, and 18.1% (95% CI, −18.3% to 43.3%) for Omicron; estimated *VE_I,p_* (vaccine effectiveness for infectiousness) was 75.3% (95% CI, 69.9% to 79.8%) for Alpha, 21.9% (95% CI, 11.0% to 31.5%) for Delta, and 18.2% (95% CI, 0.6% to 32.6%) for Omicron; and estimated *VE_T,p_* (the combined effect of direct vaccine protection and indirect vaccine effectiveness) was 94.7% (95% CI, 93.3% to 95.8%) for Alpha, 64.4% (95% CI, 58.0% to 69.8%) for Delta, and 35.8% (95% CI, 13.0% to 52.6%) for Omicron. Estimated *VE_S,p_* was also higher for Delta (68.0%; 95% CI, 62.3% to 72.8%) than Omicron (40.8%; 95% CI, 35.9% to 45.3%) for booster vaccination. Estimated *VE_S,p_* was highest for booster vaccination, followed by full vaccination and then partial vaccination, for Delta and Omicron. Including studies of all variants, for full vaccination estimated *VE_S,p_* was 61.4% (95% CI, 45.6% to 72.6%), *VE_I,p_* was 44.2% (95% CI, 20.7% to 60.8%), and *VE_T,p_* was 78.5% (95% CI, 64.8% to 86.8%).

**Table.** Estimated Vaccine Effectiveness From Household Secondary Attack Rates

Variant and Vaccination Type	Estimated vaccine effectiveness				
	*VE_I,p, _*% (95% CI)	Studies	*VE_S,p, _*% (95% CI)	Studies	*VE_T,p, _*% (95% CI)
All					
Booster vaccination	31.8 (27.1 to 36.2)	Lyngse et al,^62^ 2022; Baker et al,^63^ 2022; Jalali et al,^64^2022	49.5 (37.7 to 59.1)	Lyngse et al,^55^ 2021; Lyngse et al,^62^ 2022; Baker et al,^63^ 2022; Jalali et al,^64^2022	65.4 (55.7 to 74.9)
Full vaccination	44.2 (20.7 to 60.8)	de Gier et al,^19^ 2021; Gazit et al,^21^ 2021; Layan et al,^28^ 2021; Meyer et al,^33^ 2021; Ng et al,^37^ 2021; Singanayagam et al,^42^ 2022; Águila-Mejía et al,^61^ 2022; Baker et al,^63^ 2022; Jalali et al,^64^ 2022; Lyngse et al,^65^ 2022; de Gier et al^70^2021	61.4 (45.6 to 72.6)	de Gier et al,^19^ 2021; Gazit et al,^21^ 2021; Layan et al,^28^ 2021; Martínez-Baz et al,^32^ 2021; Ng et al,^37^ 2021; Singanayagam et al,^42^ 2022; Sachdev et al,^50^ 2021; Yi et al,^57^ 2022; Baker et al,^63^ 2022; Jalali et al,^64^ 2022; Lyngse et al,^65^ 2022; de Gier et al^70^2021	78.5 (64.8 to 86.8)
Partial vaccination	23.6 (−6.0 to 44.9)	de Gier et al,^19^ 2021; Ng et al,^37^ 2021; Singanayagam et al,^42^ 2022; Harris et al,^48^ 2021; Baker et al,^63^ 2022; Jalali et al,^64^ 2022; de Gier et al^70^2021	37.2 (16.4 to 53.0)	de Gier et al,^19^ 2021; Martínez-Baz et al,^32^ 2021; Ng et al,^37^ 2021; Singanayagam et al,^42^ 2022; Sachdev et al,^50^ 2021; Baker et al,^63^ 2022; Jalali et al^64^2022	52.1 (27.7 to 68.8)
Alpha					
Full vaccination	75.3 (69.9 to 79.8)	de Gier et al,^19^ 2021; Gazit et al,^21^ 2021; Layan et al,^28^ 2021; Meyer et al^33^2021	78.6 (76.0 to 80.9)	de Gier et al,^19^ 2021; Gazit et al,^21^ 2021; Layan et al^28^2021	94.7 (93.3 to 95.8)
Delta					
Booster vaccination	NA	NA	68.0 (62.3 to 72.8)	Lyngse et al^55^ 2021; Jalali et al^64^2022	NA
Full vaccination	21.9 (11.0 to 31.5)	Ng et al,^37^ 2021; Singanayagam et al,^42^ 2022; Águila-Mejía et al,^61^2022; Jalali et al,^64^2022; Lyngse et al,^65^ 2022; de Gier et al^70^2021	56.4 (54.6 to 58.1)	Ng et al,^37^ 2021; Singanayagam et al,^42^ 2022; Yi et al,^57^ 2022; Jalali et al,^64^ 2022; Lyngse et al,^65^ 2022; de Gier et al^70^2021	64.4 (58.0 to 69.8)
Partial vaccination	16.0 (−46.9 to 51.9)	Ng et al,^37^ 2021; Singanayagam et al,^42^ 2022; Jalali et al,^64^ 2022; de Gier et al^70^2021	37.8 (12.0 to 56.0)	Ng et al,^37^ 2021; Singanayagam et al,^42^ 2022; Yi et al,^57^ 2022; Jalali et al^64^2022	51.2 (6.1 to 74.6)
Omicron					
Booster vaccination	32.3 (25.6 to 38.3)	Lyngse et al,^62^ 2022; Baker et al,^63^ 2022; and Jalali et al^64^2022	40.8 (35.9 to 45.3)	Lyngse et al,^55^ 2021; Lyngse et al,^62^ 2022; Baker et al,^63^ 2022; and Jalali et al^64^2022	59.8 (54.7 to 64.5)
Full vaccination	18.2 (0.6 to 32.6)	Águila-Mejía et al,^61^ 2022; Baker et al,^63^ 2022; Jalali et al^64^2022	18.1 (−18.3 to 43.3)	Baker et al^63^ 2022; Jalali et al^64^2022	35.8 (13.0 to 52.6)
Partial vaccination	NA	NA	6.9 (−38.0 to 37.2)	Baker et al^63^ 2022; Jalali et al^64^2022	NA

Abbreviations: NA, not applicable because not reported in at least 2 studies; *VE_I,p_*, vaccine effectiveness for infectiousness based on the transmission probability *p*; *VE_S,p_*, vaccine effectiveness for susceptibility; *VE_T,p_*, total vaccine effectiveness.

## Discussion

We aggregated household studies to examine how variants of concern and vaccination were associated with SARS-CoV-2 household transmission rates. Full vaccination was shown to not only reduce susceptibility to infection, but also reduce transmissibility to other household contacts, albeit more so for Alpha than Delta or Omicron. SARs for Omicron, Delta, and Alpha were significantly higher than estimates for the original wild-type variant.

We found evidence of reduced infectiousness from breakthrough cases among fully vaccinated index cases compared with unvaccinated, though the level of protection conferred for Delta (*VE_I,p_* = 21.9%) and Omicron (18.2%) was lower than for Alpha (75.3%). These findings are consistent with a cohort study^71^ in England, which demonstrated a reduction in estimated vaccine effectiveness against onward transmission for Omicron compared with Delta in household and nonhousehold settings. That study did show, however, that infectiousness was reduced from booster-vaccinated individuals for both Delta and Omicron cases, but less so for Omicron. An observational cohort study^72^ from England, which included contacts outside the household, also reported that 2 doses of BNT162b2 or ChAdOx1 reduced onward transmission of Delta less than Alpha, and the protection of vaccination against onward transmission waned over time. Our 2-dose *VE_I,p_* estimate of 75.3% for Alpha was similar to the *VE_I_* of 72.1% (95% CI, 36.6%-89.3%) based on adjusted odds ratios reported by Hayek et al^73^ during a period in which the Alpha variant was dominant. Potential mechanisms for reduced infectiousness following vaccination for Alpha include decreases in the respiratory tract viral load, duration of infection, and severity of symptoms.^74^ Our overall 2-dose *VE_I,p_* estimate of 44.2% was within the lower range reported for *VE_I_* (41%-79%) from a modeling study that used household data from Israel before Delta or Omicron became widespread.^75^

Fully vaccinated contacts were generally less susceptible to infection with Alpha and Delta than unvaccinated contacts, and individuals who were booster vaccinated were less susceptible to Omicron. Our 3-dose *VE_S,p_* estimate of 40.8% for Omicron is closer to the greater than 60-day postbooster estimate (47.4%; 95% CI, 40.5%–53.5%) than the 14- to 60-day estimate (71.6%; 95% CI, 69.7%-73.4%) reported in a test-negative design study^76^ of mRNA-1273, which adjusted for age, sex, race and ethnicity, and specimen collection date. Our 2-dose *VE_S,p_* estimate of 56.4% for Delta is also lower than the 61.3% (95% CI, 55.1%-66.7%) at greater than 270 days.^76^ Booster doses of either BNT162b2 or mRNA-1273 increased direct protection against mild Omicron infection, but that protection waned over time.^77^ Lower protection against susceptibility for Omicron may be attributed to variations in the spike glycoprotein and its ability to evade immune responses.^78^ Less severe symptoms for Omicron may also lead to reduced household vigilance in maintaining isolation of the infected individual. One study^62^ included in this analysis reported higher susceptibility to BA.2 compared with BA.1 among unvaccinated, fully vaccinated, and booster-vaccinated individuals, which may be attributed to higher viral load. Other observational studies conducted before the emergence of Omicron demonstrated reduced susceptibility to infection among high-risk or household contacts vaccinated with BNT162b2 or ChAdOx1 in Scotland,^79^ BNT162b2 in Sweden,^80^ and BNT162b2 or mRNA-1273 in Belgium.^81^ Studies have reported that full vaccination with mRNA vaccines or ChAdOx1 effectively prevent infection against the original wild-type, Alpha, and Beta variants, but are less protective against infection from Delta.^82,83^ Additionally, there is a combined net protective effect from both the index cases and contacts being fully vaccinated as demonstrated by our overall estimate of *VE_T,p_* (78.5%). Our overall estimates for full vaccination of *VE_S,p_* (61.4%) and *VE_T,p_* were lower than the age-adjusted *VE_S _*(80.5%, 95% CI, 78.9%-82.1%) and *VE_T _*(88.5%, 95% CI, 82.3%-94.8%) reported by Prunas et al*.*^84^

SAR estimates for Omicron (42.7%), Alpha (36.4%), and Delta (29.7%) variants were higher than the overall SARs previously reported (18.9%)^1^ for study periods earlier in the pandemic when the wild-type variant was prevalent. Public Health England (PHE), which tracks SARs for variants of concern and variants of interest regardless of vaccination status for index cases and household contacts, found that almost all current household transmission is from Omicron BA.2 and BA.1 with increasing prevalence of BA.2.^85^ PHE had previously reported similar SARs for Alpha (10.2%; 95% CI, 10.1%-10.3%) and Delta (10.4%; 95% CI, 10.4%-10.5%) variants,^86^ noting however that direct comparisons between variants are not valid, as vaccination levels and social restrictions in England have varied over this period.

### Limitations

This study has limitations that should be addressed. There was large heterogeneity in SARs over time, which may be attributed to variations in study methods, environmental factors, and contact patterns. Myriad factors preclude our ability to make direct comparisons of vaccine effectiveness across studies, including differences in the study population (eg, age, comorbidities, and serostatus), location, diagnostic procedures and tools, definition of vaccination status (eg, time elapsed since vaccination or dosage) (eTable 8 in the Supplement), follow-up duration, viral variants, vaccine types and coverage rates, intensity of the epidemic, community behavior, and use of nonpharmaceutical interventions (masks and social distancing).^87^ For example, in this analysis Singanayagam et al^42^ included households of any size with contacts 5 or more years, whereas Gazit et al^21^ restricted to households with only 1 contact other than the index case. Moreover, Ng et al^37^ in Singapore reported that all identified close contacts were placed under a legally binding quarantine for 14 days during which they were not allowed to leave their homes, whereas contacts in other studies may have had a higher risk of infection outside the household. Few studies disaggregated SARs by both vaccination status of the index cases and contacts. We were unable to calculate *VE_T,p_* where both the index cases and contacts were fully vaccinated compared with those where both the index cases and contacts were unvaccinated (*VE_T,p_* = 1 − *SAR*_11_ / *SAR*_00_) as there were too few studies that reported this information. The studies included in this review are from contact tracing investigations, which are more likely to identify symptomatic index cases than asymptomatic individuals and which could inflate the crude SAR. This may also underestimate the reduction in transmission from vaccination for people infected with Delta.^88^ There were insufficient data to evaluate Omicron subvariants BA.1 and BA.2 separately and to determine vaccine effectiveness for specific subgroups (eg, by age group).

## Conclusions

This meta-analysis of 135 studies suggests that there is increased transmissibility of emerging SARS-CoV-2 variants of concern in the confines of the household where there is prolonged close contact between household members and index cases. Full vaccination reduced susceptibility and infectiousness, but more so for Alpha than Delta and Omicron. The changes in estimated vaccine effectiveness underscore the challenges of developing effective vaccines concomitant with viral evolution.

## Notes

Supplement. **eFigure 1.** PRISMA Flow Diagram **eFigure 2.** Funnel Plots of Studies Reporting Household Secondary Attack Rates With Midpoints in 2021 and Through April 2020 **eFigure 3.** Funnel Plots of Studies Reporting Household Secondary Attack Rates for Alpha (B.1.1.7) and Delta (B.1.617.2) Variants **eFigure 4.** Household Secondary Attack Rates for Omicron (B.1.1.529), Alpha (B.1.1.7), Delta (B.1.617.2), and Beta (B.1.351) Variants From Unvaccinated Index Cases to Unvaccinated Household Contacts **eFigure 5.** Household Secondary Attack Rates by Index Case Vaccination Status With All Contacts Are Included Regardless of Vaccination Status **eFigure 6.** Funnel Plots of Studies Reporting Household Secondary Attack Rates From Unvaccinated or Fully Vaccinated Index Cases to All Contacts Regardless of Vaccination Status **eFigure 7.** Household Secondary Attack Rates by Index Case Vaccination Status With Only Unvaccinated Contacts Included **eFigure 8.** Household Secondary Attack Rates by Contact Vaccination Status With All Index Cases Included Regardless of Vaccination Status **eFigure 9.** Funnel Plots of Studies Reporting Household Secondary Attack Rates to Unvaccinated or Fully Vaccinated Contacts From All Index Cases Regardless of Vaccination Status **eFigure 10.** Household Secondary Attack Rates by Contact Vaccination Status With Only Unvaccinated Index Cases Included **eFigure 11.** Household Secondary Attack Rates by Vaccination Status of the Index Cases and Contacts **eTable 1.** Electronic Databases and Search Strategy for Household Secondary Attack Rate of SARS-CoV-2 **eTable 2.** Description of Studies Identified From June 18, 2021 to March 8, 2022 **eTable 3. **References for Studies Included in Figure 1 of SAR Over Time **eTable 4. **Risk of Bias Assessment for Studies Included in Review of Household Transmissibility of SARS-CoV-2 Using the Same Modified Version of the Newcastle–Ottawa Quality Assessment Scale for Observational Studies Used by Fung et al **eTable 5. **Pairwise Analyses of Index Case Vaccination Status Using Only Studies in Which SARs Were Reported From Both Relevant Subgroups **eTable 6. **Pairwise Analyses of Household Contact Vaccination Status Using Only Studies in Which SARs Were Reported From Both Relevant Subgroups **eTable 7. **Household Secondary Attack Rates by Vaccine Type and Contact Vaccination Status With All Index Cases Included Regardless of Vaccination Status **eTable 8. **Vaccination Status Definitions for Studies That Reported Household Secondary Attack Rates by Vaccination Status of Index Cases or Contacts
eReferences
