# Supplementary material for: Household Secondary Attack Rates of SARS-CoV-2 by Variant and Vaccination Status: An Updated Systematic Review and Meta-analysis
Source: JAMA Netw Open. 2022 Apr 28;5(4):e229317. doi: 10.1001/jamanetworkopen.2022.9317 (PMC9051991; doi:10.1001/jamanetworkopen.2022.9317)
Supplement: Supplement. — eFigure 1. PRISMA Flow Diagram eFigure 2. Funnel Plots of Studies Reporting Household Secondary Attack Rates With Midpoints in 2021 and Through April 2020 eFigure 3. Funnel Plots of Studies Reporting Household Secondary Attack Rates for Alpha (B.1.1.7) and Delta (B.1.617.2) Variants eFigure 4. Household Secondary Attack Rates for Omicron (B.1.1.529), Alpha (B.1.1.7), Delta (B.1.617.2), and Beta (B.1.351) Variants From Unvaccinated Index Cases to Unvaccinated Household Contacts eFigure 5. Household Secondary Attack Rates by Index Case Vaccination Status With All Contacts Are Included Regardless of Vaccination Status eFigure 6. Funnel Plots of Studies Reporting Household Secondary Attack Rates From Unvaccinated or Fully Vaccinated Index Cases to All Contacts Regardless of Vaccination Status eFigure 7. Household Secondary Attack Rates by Index Case Vaccination Status With Only Unvaccinated Contacts Included eFigure 8. Household Secondary Attack Rates by Contact Vaccination Status With All Index Cases Included Regardless of Vaccination Status eFigure 9. Funnel Plots of Studies Reporting Household Secondary Attack Rates to Unvaccinated or Fully Vaccinated Contacts From All Index Cases Regardless of Vaccination Status eFigure 10. Household Secondary Attack Rates by Contact Vaccination Status With Only Unvaccinated Index Cases Included eFigure 11. Household Secondary Attack Rates by Vaccination Status of the Index Cases and Contacts eTable 1. Electronic Databases and Search Strategy for Household Secondary Attack Rate of SARS-CoV-2 eTable 2. Description of Studies Identified From June 18, 2021 to March 8, 2022 eTable 3. References for Studies Included in Figure 1 of SAR Over Time eTable 4. Risk of Bias Assessment for Studies Included in Review of Household Transmissibility of SARS-CoV-2 Using the Same Modified Version of the Newcastle–Ottawa Quality Assessment Scale for Observational Studies Used by Fung et al eTable 5. Pairwise Analyses of Index Case Vaccination Status Us [file jamanetwopen-e229317-s001.pdf]

Supplemental Online Content

Madewell ZJ, Yang Y, Longini IM Jr, Halloran ME, Dean NE. Household secondary attack rates of SARS-CoV-2 by variant and vaccination status: an updated systematic review and meta-analysis. *JAMA Netw Open*. 2022;5(4):e229317. doi:10.1001/jamanetworkopen.2022.9317

eFigure 1. PRISMA Flow Diagram
eFigure 2. Funnel Plots of Studies Reporting Household Secondary Attack Rates With Midpoints in 2021 and Through April 2020
eFigure 3. Funnel Plots of Studies Reporting Household Secondary Attack Rates for Alpha (B.1.1.7) and Delta (B.1.617.2) Variants
eFigure 4. Household Secondary Attack Rates for Omicron (B.1.1.529), Alpha (B.1.1.7), Delta (B.1.617.2), and Beta (B.1.351) Variants From Unvaccinated Index Cases to Unvaccinated Household Contacts
eFigure 5. Household Secondary Attack Rates by Index Case Vaccination Status With All Contacts Are Included Regardless of Vaccination Status
eFigure 6. Funnel Plots of Studies Reporting Household Secondary Attack Rates From Unvaccinated or Fully Vaccinated Index Cases to All Contacts Regardless of Vaccination Status
eFigure 7. Household Secondary Attack Rates by Index Case Vaccination Status With Only Unvaccinated Contacts Included
eFigure 8. Household Secondary Attack Rates by Contact Vaccination Status With All Index Cases Included Regardless of Vaccination Status
eFigure 9. Funnel Plots of Studies Reporting Household Secondary Attack Rates to Unvaccinated or Fully Vaccinated Contacts From All Index Cases Regardless of Vaccination Status
eFigure 10. Household Secondary Attack Rates by Contact Vaccination Status With Only Unvaccinated Index Cases Included
eFigure 11. Household Secondary Attack Rates by Vaccination Status of the Index Cases and Contacts
eTable 1. Electronic Databases and Search Strategy for Household Secondary Attack Rate of SARS-CoV-2
eTable 2. Description of Studies Identified From June 18, 2021 to March 8, 2022
eTable 3. References for Studies Included in Figure 1 of SAR Over Time
eTable 4. Risk of Bias Assessment for Studies Included in Review of Household Transmissibility of SARS-CoV-2 Using the Same Modified Version of the Newcastle–Ottawa Quality Assessment Scale for Observational Studies Used by Fung et al
eTable 5. Pairwise Analyses of Index Case Vaccination Status Using Only Studies in Which SARs Were Reported From Both Relevant Subgroups
eTable 6. Pairwise Analyses of Household Contact Vaccination Status Using Only Studies in Which SARs Were Reported From Both Relevant Subgroups

© 2022 Madewell ZJ et al. *JAMA Network Open.*

eTable 7. Household Secondary Attack Rates by Vaccine Type and Contact Vaccination Status With All Index Cases Included Regardless of Vaccination Status
eTable 8. Vaccination Status Definitions for Studies That Reported Household Secondary Attack Rates by Vaccination Status of Index Cases or Contacts
eReferences

This supplemental material has been provided by the authors to give readers additional information about their work.

© 2022 Madewell ZJ et al. *JAMA Network Open.*

eFigure 1. PRISMA Flow Diagram

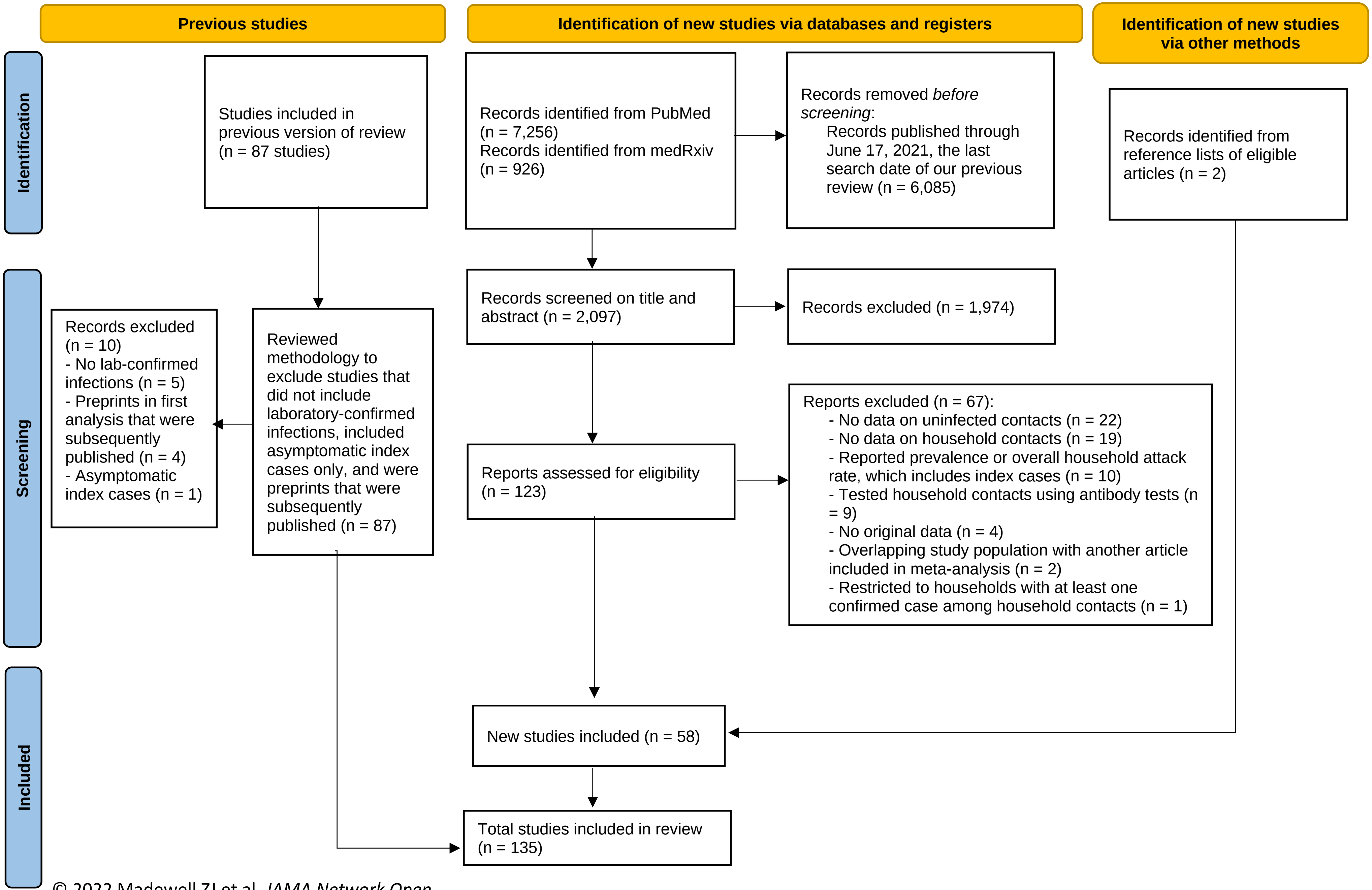

© 2022 Madewell ZJ et al. *JAMA Network Open.*

eFigure 2. Funnel Plots of Studies Reporting Household Secondary Attack Rates With Midpoints in 2021 and Through April 2020

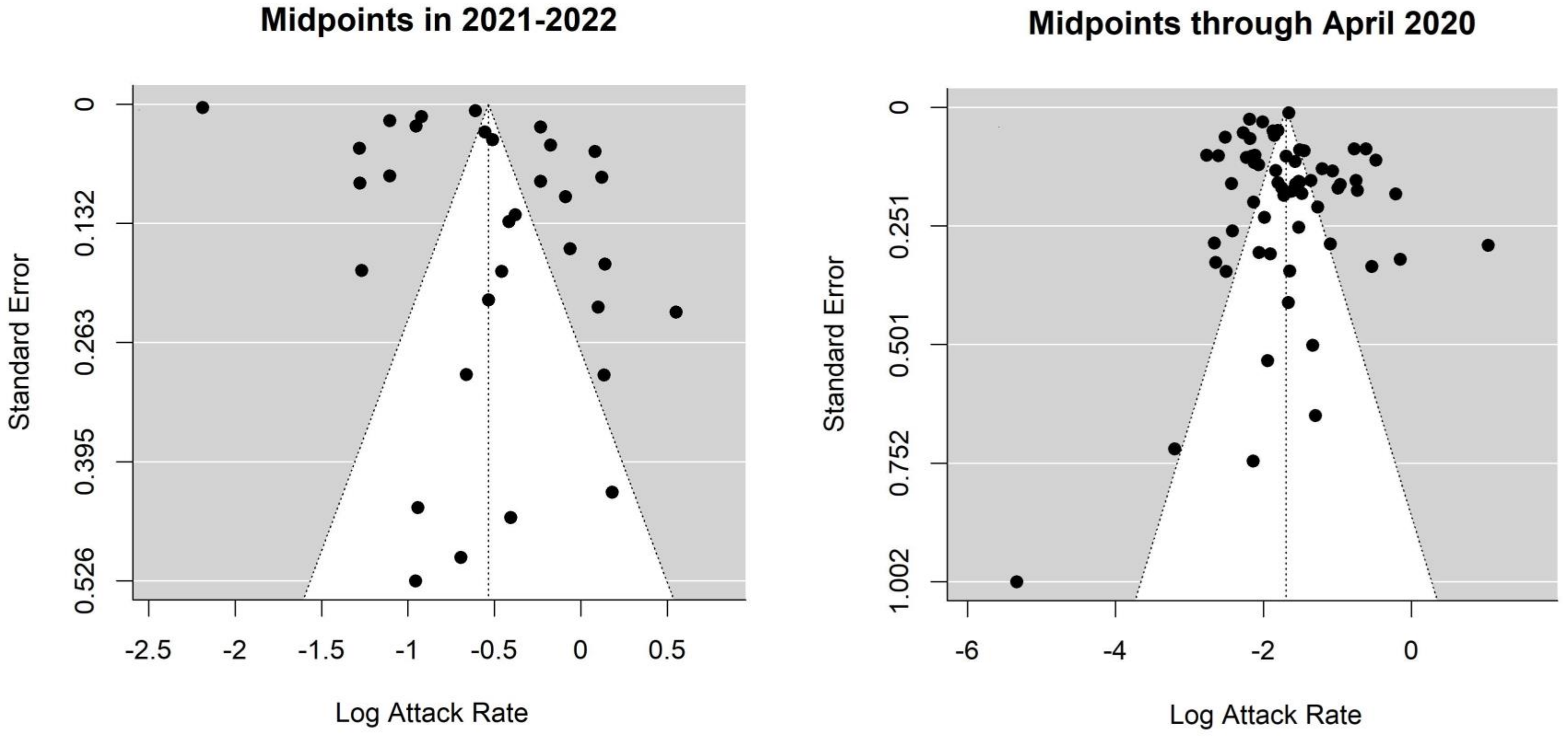

Begg & Mazumdar rank correlation test: studies with midpoints in 2021-2022 (*P*<0.001); midpoints through April 2020 (*P*=0.508). Excluding one study in 2021[1] that had a relatively low SAR of 10.1% improved the funnel plot symmetry and resulted in a SAR of 38.6% (95%CI, 34.5%-42.8%) for studies with midpoints in 2021-2022.

© 2022 Madewell ZJ et al. *JAMA Network Open.*

eFigure 3. Funnel Plots of Studies Reporting Household Secondary Attack Rates for Alpha (B.1.1.7) and Delta (B.1.617.2) Variants

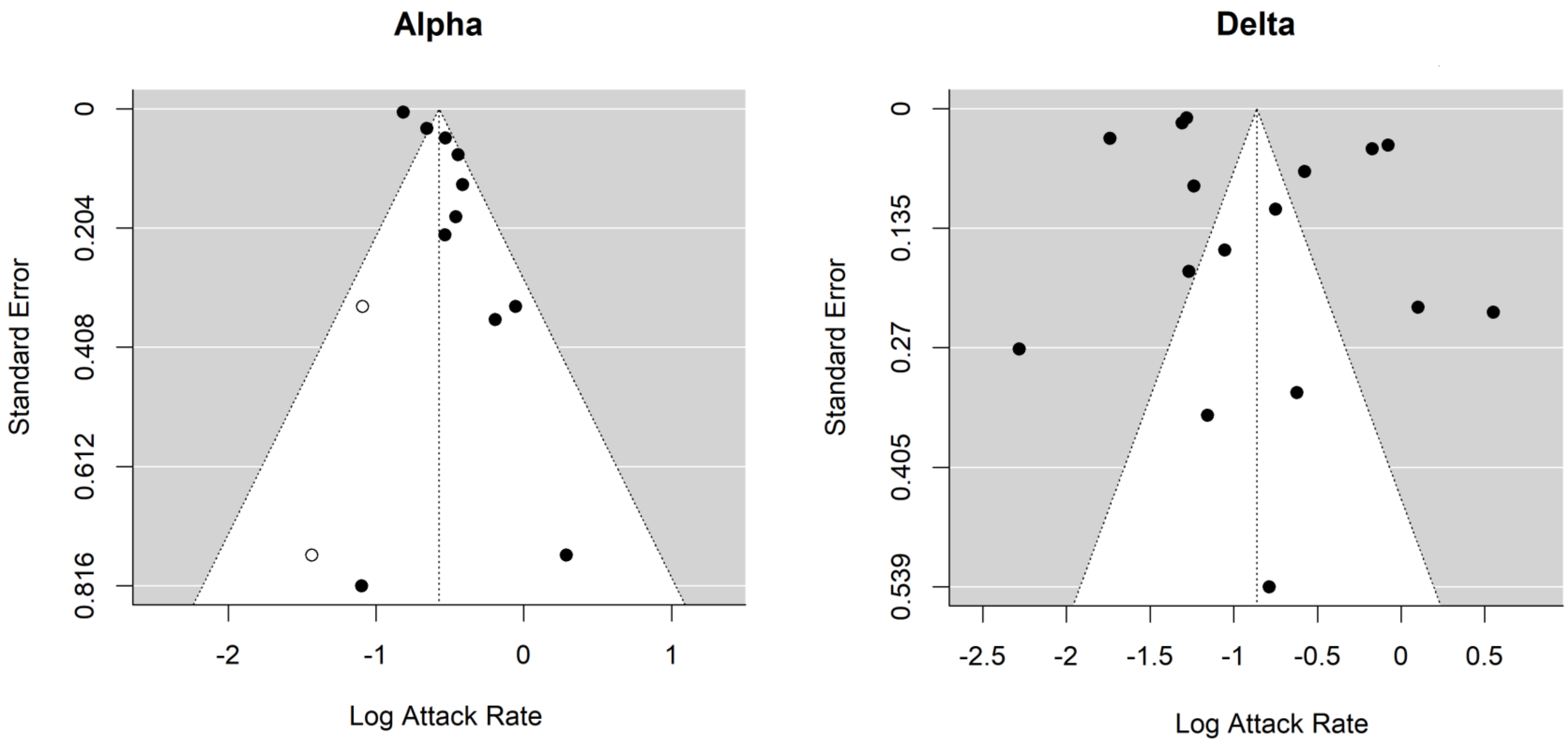

Begg & Mazumdar rank correlation test: Alpha (pre-imputation) (*P*=0.087); Delta (*P*=0.894)

© 2022 Madewell ZJ et al. *JAMA Network Open.*

eFigure 4. Household Secondary Attack Rates for Omicron (B.1.1.529), Alpha (B.1.1.7), Delta (B.1.617.2), and Beta (B.1.351) Variants From Unvaccinated Index Cases to Unvaccinated Household Contacts

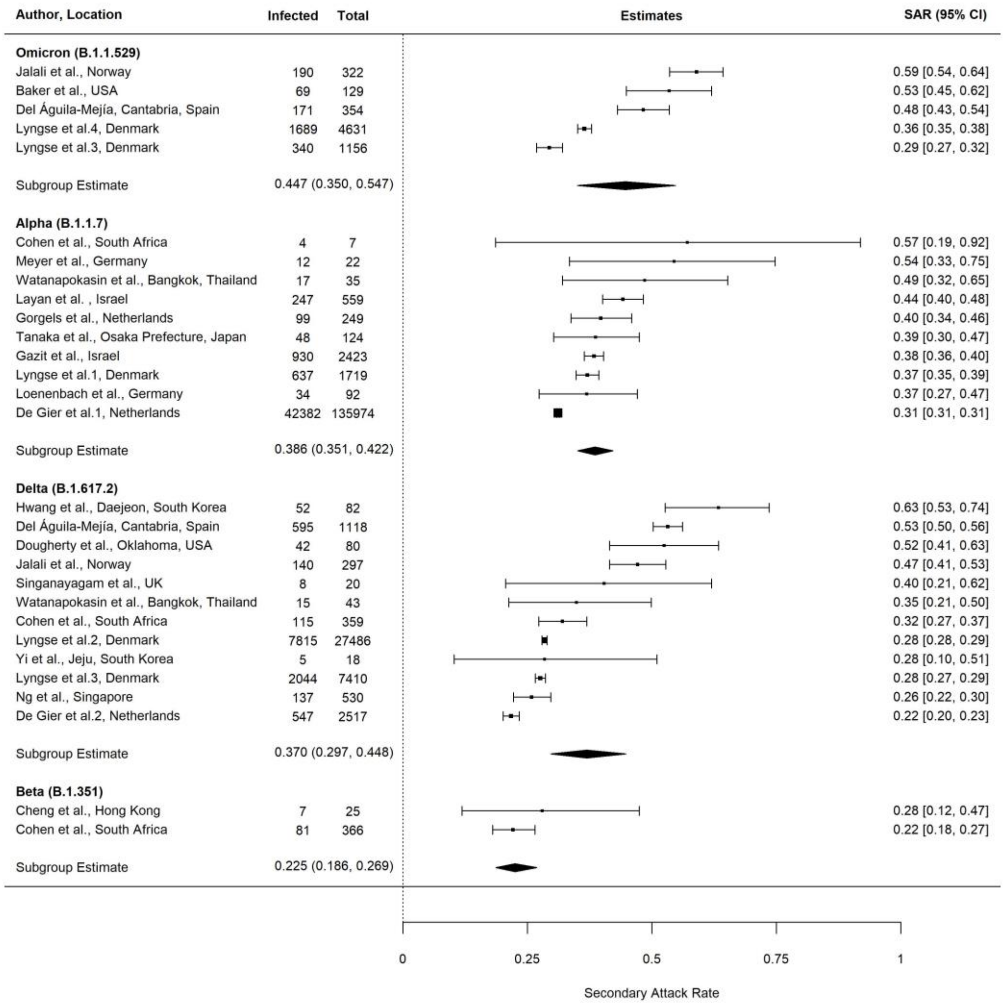

Three studies included did not disaggregate household secondary attack rates by vaccination status. Watanapokasin *et al*. reported that 83% of index cases were unvaccinated. Vaccination status was not provided for contacts. Dougherty *et al.* reported that only 17 of 194 (8.8%) exposed individuals in the study were fully vaccinated. Cohen *et al.* reported that vaccine uptake was low in study sites reaching 5% fully vaccinated by the end of follow up. Point sizes are an inverse function of the precision of the estimates, and bars correspond to 95%CIs. Diamonds represent summary SAR estimates with corresponding 95%CIs.

© 2022 Madewell ZJ et al. *JAMA Network Open.*

eFigure 5. Household Secondary Attack Rates by Index Case Vaccination Status With All Contacts Are Included Regardless of Vaccination Status

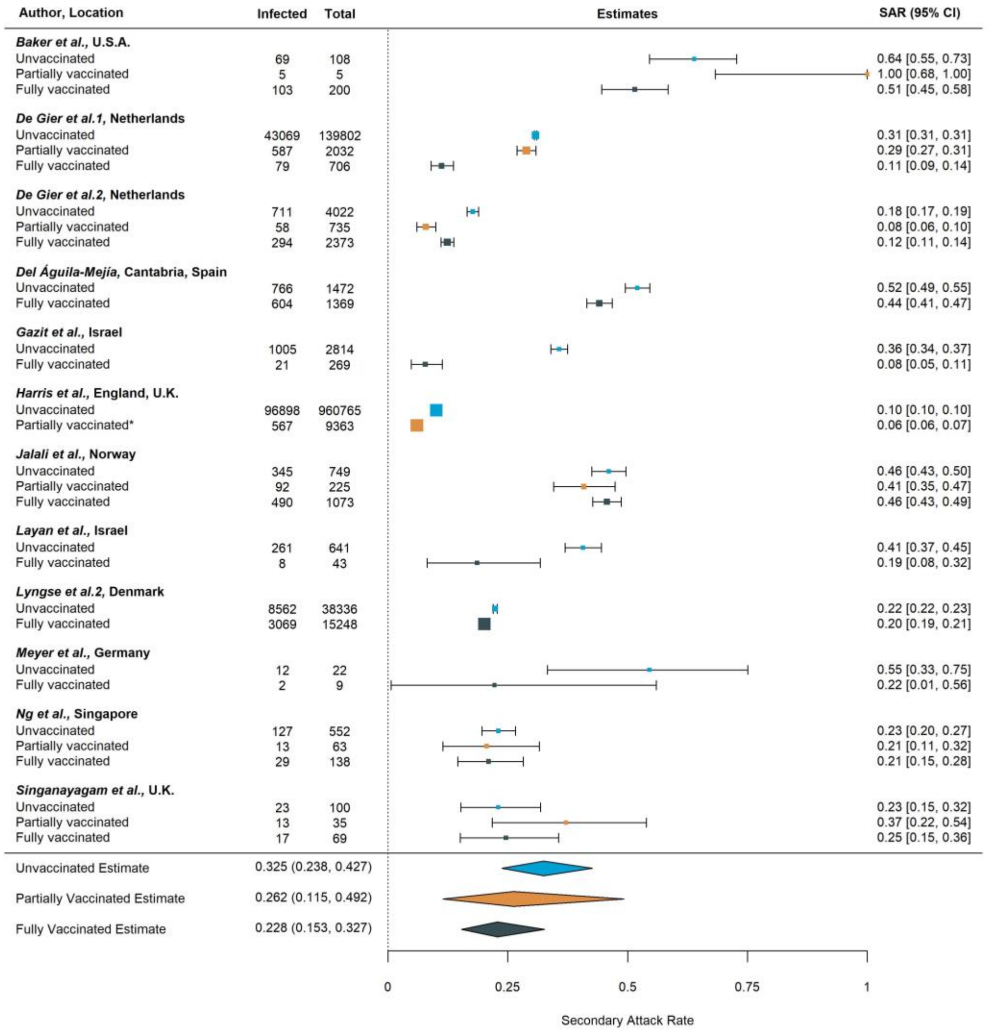

Point sizes are an inverse function of the precision of the estimates, and bars correspond to 95%CIs. Diamonds represent summary SAR estimates with corresponding 95%CIs.

© 2022 Madewell ZJ et al. *JAMA Network Open.*

eFigure 6. Funnel Plots of Studies Reporting Household Secondary Attack Rates From Unvaccinated or Fully Vaccinated Index Cases to All Contacts Regardless of Vaccination Status

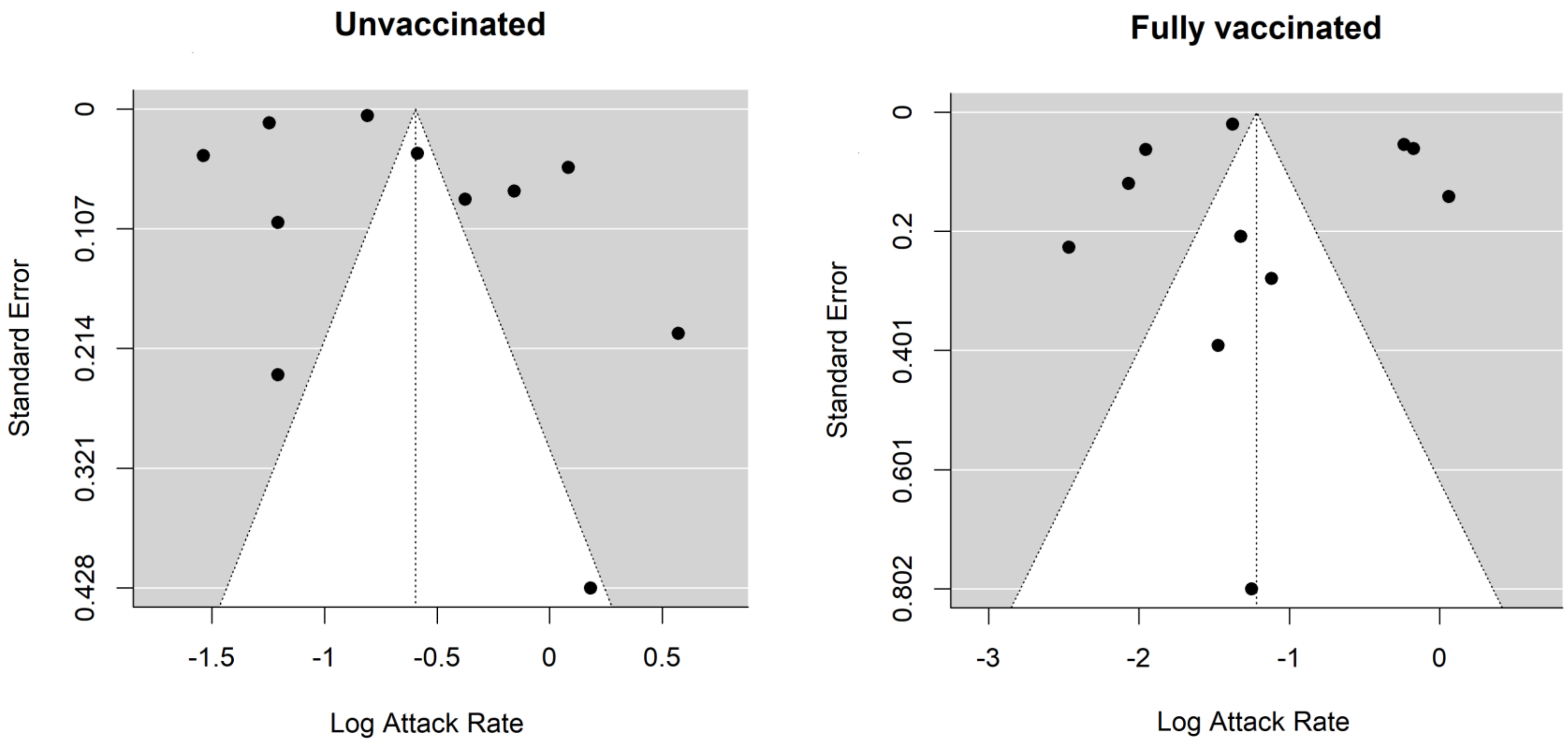

Begg & Mazumdar rank correlation test: unvaccinated (*P*=0.542); fully vaccinated (*P*=0.879)

© 2022 Madewell ZJ et al. *JAMA Network Open.*

eFigure 7. Household Secondary Attack Rates by Index Case Vaccination Status With Only Unvaccinated Contacts Included

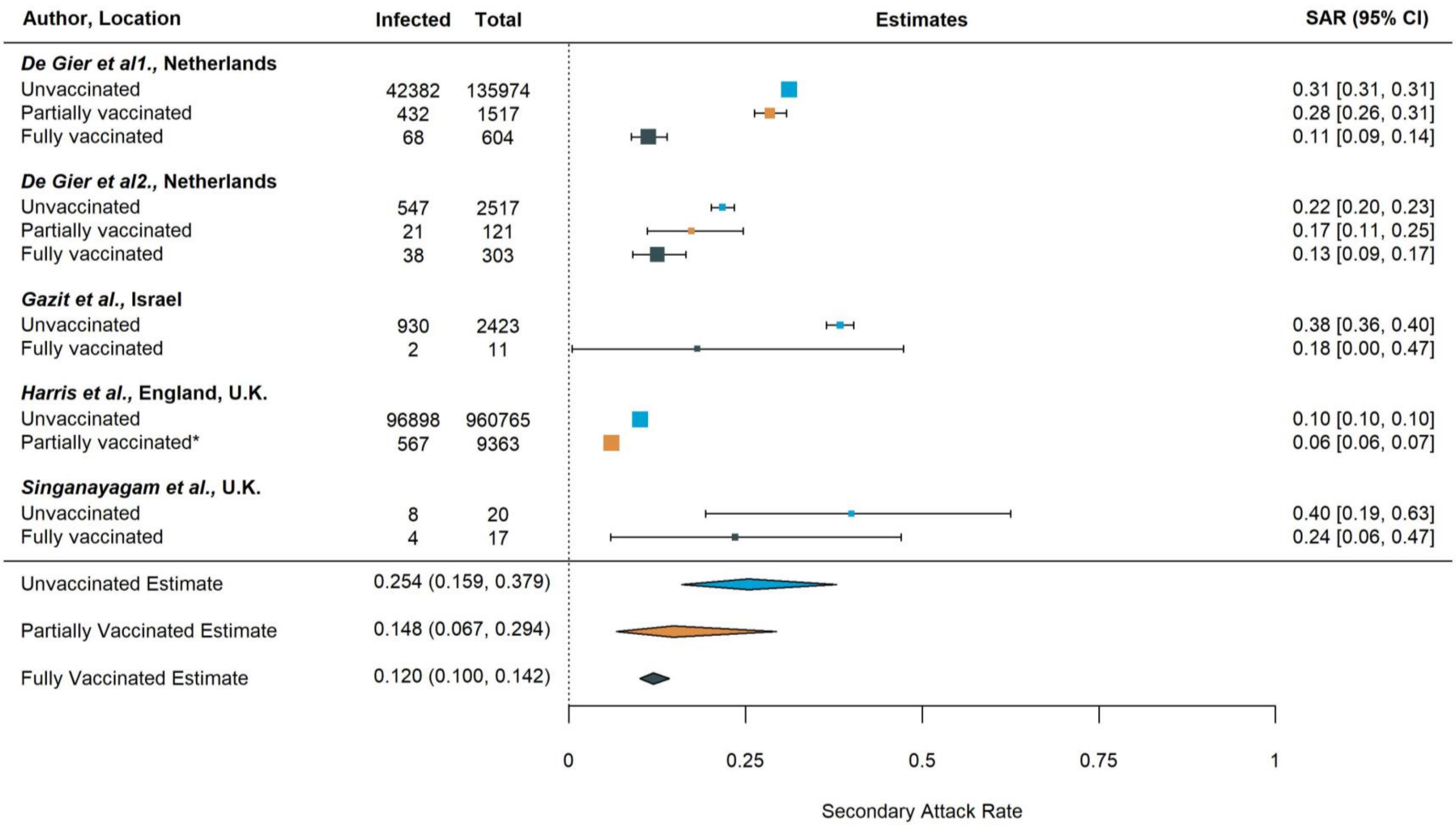

*For *Harris et al.*, most of the vaccinated index cases (93%) had received only the first dose of vaccine and secondary attack rates were not disaggregated by dose. Point sizes are an inverse function of the precision of the estimates, and bars correspond to 95%CIs. Diamonds represent summary SAR estimates with corresponding 95%CIs.

© 2022 Madewell ZJ et al. *JAMA Network Open.*

eFigure 8. Household Secondary Attack Rates by Contact Vaccination Status With All Index Cases Included Regardless of Vaccination Status

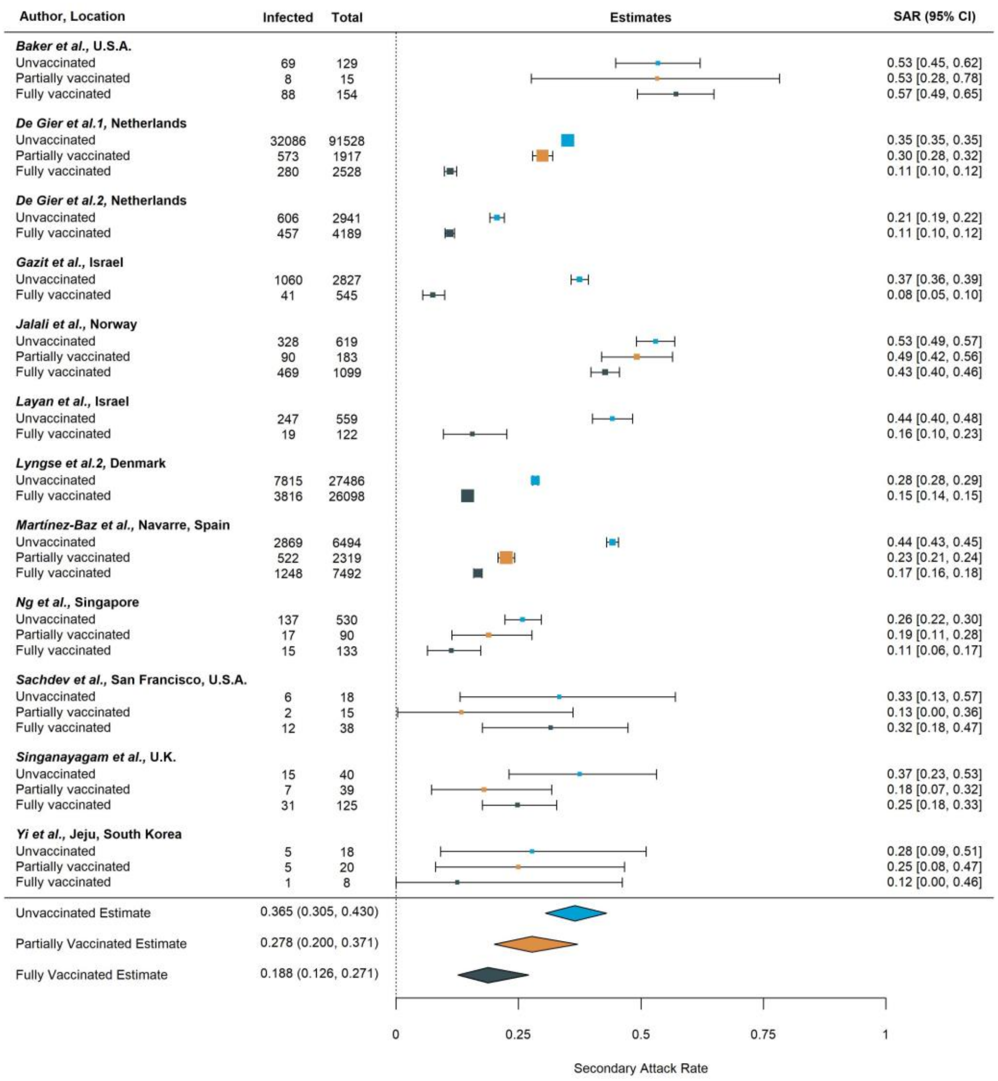

Point sizes are an inverse function of the precision of the estimates, and bars correspond to 95%CIs. Diamonds represent summary SAR estimates with corresponding 95%CIs.

© 2022 Madewell ZJ et al. *JAMA Network Open.*

eFigure 9. Funnel Plots of Studies Reporting Household Secondary Attack Rates to Unvaccinated or Fully Vaccinated Contacts From All Index Cases Regardless of Vaccination Status

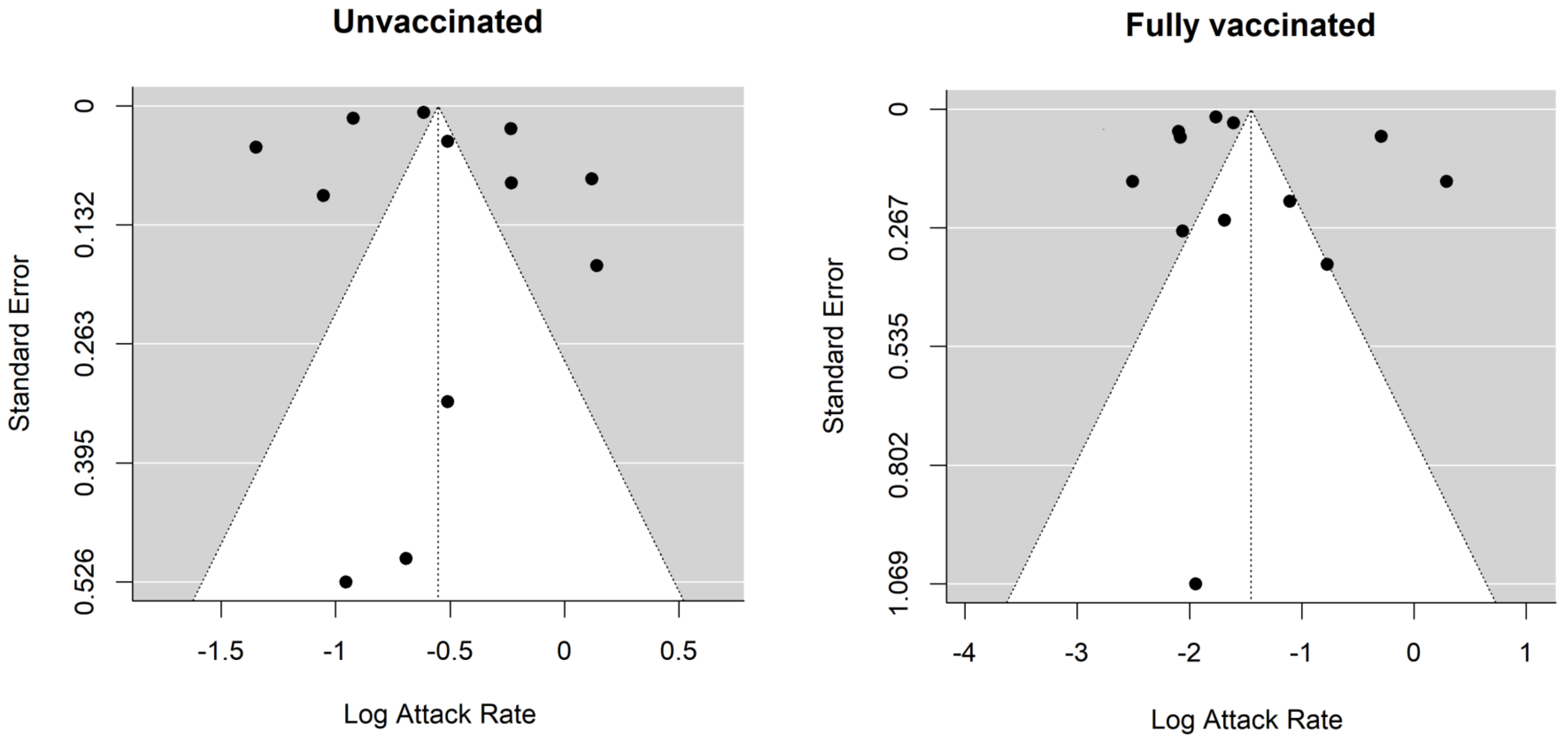

Begg & Mazumdar rank correlation test: unvaccinated (*P*=0.311); fully vaccinated (*P*=0.638)

© 2022 Madewell ZJ et al. *JAMA Network Open.*

eFigure 10. Household Secondary Attack Rates by Contact Vaccination Status With Only Unvaccinated Index Cases Included

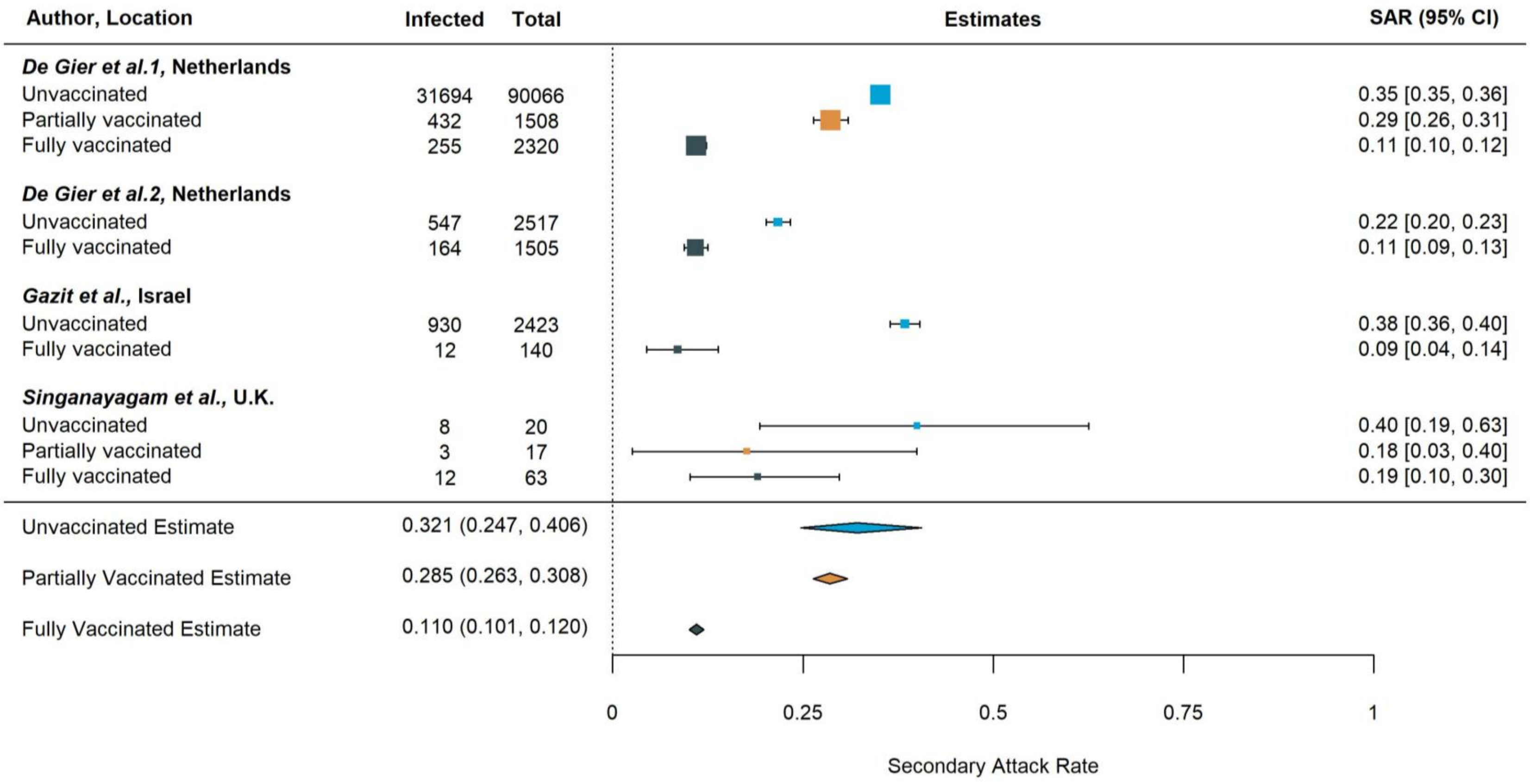

Point sizes are an inverse function of the precision of the estimates, and bars correspond to 95%CIs. Diamonds represent summary SAR estimates with corresponding 95%CIs.

© 2022 Madewell ZJ et al. *JAMA Network Open.*

eFigure 11. Household Secondary Attack Rates by Vaccination Status of the Index Cases and Contacts

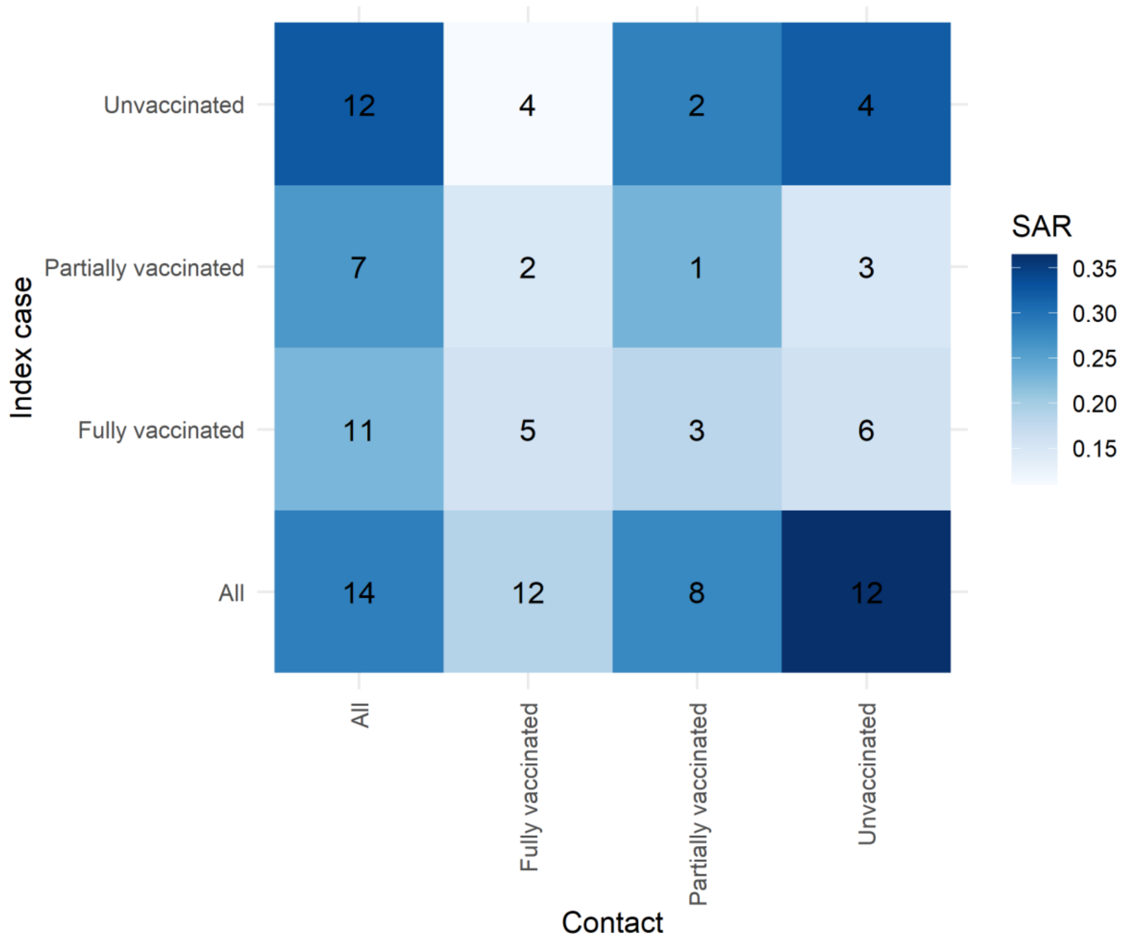

The number of studies included in each analysis is shown.

© 2022 Madewell ZJ et al. *JAMA Network Open.*

eTable 1. Electronic Databases and Search Strategy for Household Secondary Attack Rate of SARS-CoV-2

Database: PubMed: 1,791 retrieved articles; medRxiv: 306 retrieved		
#1: "SARS-CoV-2" [All Fields] OR "COVID-19" [All Fields] OR "severe acute respiratory syndrome" [All Fields] OR "SARS" [All Fields] OR "SARS-CoV" [All Fields] OR "coronavirus" [All Fields] OR "variant" [All Fields] OR "vaccination" [All Fields] or “immunization” [All Fields]	#2: "secondary attack rate" [All Fields] OR "secondary infection rate" [All Fields] OR "household" [All Fields] OR "family contacts" [All Fields] OR "close contacts" [All Fields] OR "index case" [All Fields] OR "contact transmission" [All Fields] OR "contact attack rate" [All Fields] OR "family transmission" [All Fields]	"2021/06/18"[Date - Publication]: "3000"[Date - Publication]
**#1 AND #2 AND #3**		

© 2022 Madewell ZJ et al. *JAMA Network Open.*

eTable 2. Description of Studies Identified From June 18, 2021 to March 8, 2022

Authors	Location	Study period	No. index cases	Index case symptom status	Duration of follow-up (days)	Test used to diagnose contacts	Universal testing or only symptomatic	Number of tests per contact	Household SAR (infected/total)
Afonso et al.[2]	Goiânia, Brazil	June 15 – October 28, 2020	187	Symptomatic and asymptomatic	14	RT-PCR	Universal	1	Untyped: 25.1% (67/267)
Baker et al.[3]	USA	November 2021 – February 2022	183	Symptomatic and asymptomatic	7	Nucleic acid amplification test or antigen test	Universal	1	Omicron: 52.7% (227/431)
Bistaraki et al.[4]	Greece	October 1 – December 9, 2020	–	Symptomatic and asymptomatic	–	–	–	–	Untyped: 18.3% (10,247/55,991)
Burke et al.[5]	USA	January 14 – April 4, 2020	69	Symptomatic	14	Symptom-based diagnosis	Symptomatic	0	Untyped: 32% (62/193)
Calvani et al.[6]	Rome, Italy	October 16 –December 19, 2020	28	Symptomatic and asymptomatic	–	–	Universal	1	Untyped: 30.6% (22/72)
Cheng et al.[7]	Hong Kong	April – May, 2021	–	Symptomatic and asymptomatic	14 or 21	RT-PCR and whole genome sequencing	Universal	4	Beta: 28.0% (7/25)
Chu et al.[8]	Georgia, USA	July 17 – August 24, 2020	224	Symptomatic and asymptomatic	–	Antigen test	Universal	At least 1	Untyped: 12.2% (46/377)
Clifford et al.[9]	England	February 2, 2021 – September 10, 2021	195	Symptomatic and asymptomatic	14	RT-PCR & genomic sequencing	Universal	3	Alpha: 49% (95% CI: 34-63%)<br>Delta: 81% (95% CI: 57%-96%)<br>Numerator/denominator provided for overall only: 40.6% (113/278)
Cohen et al.[10]	South Africa	July 16, 2020 –	103	Symptomatic and asymptomatic	14	RT-PCR	Universal	Multiple	Alpha: 57.1% (4/7)<br>Beta: 22.1% (81/366)<br>Delta: 32.0% (115/359)

© 2022 Madewell ZJ et al. *JAMA Network Open.*

Authors	Location	Study period	No. index cases	Index case symptom status	Duration of follow-up (days)	Test used to diagnose contacts	Universal testing or only symptomatic	Number of tests per contact	Household SAR (infected/total)
		March 31, 2021							Wild type: 10.5% (16/153)
De Gier et al.[11]	Netherlands	February 1 – May 27, 2021	85,210	Symptomatic	14	RT-PCR, loop mediated isothermal amplification or antigen test	Universal	–	Untyped: 30.7% (43,735/142,540)
De Gier et al.[12]	Netherlands	August 9 – September 24, 2021	4,921	Symptomatic	10	RT-PCR, loop mediated isothermal amplification or antigen test	Universal	2	Untyped: 14.9% (1,063/7,130)
Del Águila-Mejía et al.[13]	Cantabria, Spain	December 2021	622	Symptomatic and asymptomatic	9	RT-PCR and whole-genome sequencing	Universal	2	Omicron: 49.3% (263/533)<br>Delta: 48.0% (1129/2350)
Dougherty et al.[14]	Oklahoma, U.S.A.	April 15 – May 3, 2021	21	Symptomatic and asymptomatic	14	Sequencing	Universal	1	Delta: 52.5% (42/80)
Dub et al.[15]	Helsinki, Finland	March 24, 2020 – June 17, 2020	39	Symptomatic and asymptomatic	28	RT-PCR	Universal	4	Untyped: RT-PCR: 32.2% (28/87)
Friedman-Klabanoff et al.[16]	U.S.A.	March – August 2020	–	Symptomatic and asymptomatic	14	RT-PCR	Universal	14	Untyped: 15.2% (86/567)

© 2022 Madewell ZJ et al. *JAMA Network Open.*

Authors	Location	Study period	No. index cases	Index case symptom status	Duration of follow-up (days)	Test used to diagnose contacts	Universal testing or only symptomatic	Number of tests per contact	Household SAR (infected/total)
Gazit et al.[17]	Israel	December 20, 2020 – March 17, 2021	2,827	Symptomatic and asymptomatic	10	RT-PCR	Universal	1	Untyped: 34.1% (1,373/4,024)
Ge et al.[18]	Zhejiang Province, China	January 8 – July 30, 2020	370	Symptomatic and asymptomatic	14	RT-PCR	Universal	Multiple	Untyped: 10.1% (260/2,565)
Gorgels et al.[19]	Netherlands	March – April, 2021	97	Symptomatic and asymptomatic	14	RT-PCR or antigen test	Universal	At least 1	Alpha: 39.8% (99/249)
Hwang et al.[20]	Daejeon, South Korea	June 22 – July 31, 2021	23	Symptomatic and asymptomatic	14	RT-PCR	Universal	3	Delta: 64.2% (52/82).
Jagdale et al.[21]	Pune City, India	April 1 – May 15, 2020	119	Symptomatic and asymptomatic	14	RT-PCR	Universal	1	Untyped: 35% (198/565)
Jalali et al.[22]	Norway	December 14, 2021 – January 23, 2022	1122	Symptomatic and asymptomatic	10	RT-PCR or whole genome sequencing	Universal	–	1299 contacts * 0.41 SAR = 662<br>Omicron: 41.0% (662/1299)<br>870 contacts * 0.36 SAR = 313<br>Delta: 36.0% (313/870)
Kang et al.[23]	Guangdong, China	May 21 – June 18, 2021	73	Symptomatic and asymptomatic	14	RT-PCR	Universal	Multiple	Delta: 22.0% (38/173)
Karumanagoundar et al.[24]	Tamil Nadu, India	March 14, 2020 – May 30, 2020	931	Symptomatic and asymptomatic	14	RT-PCR	Universal	1	Untyped: 13.4% (464/3,474)
Layan et al.[25]	Israel	December 31 2020 – April 26 2021	212	Symptomatic and asymptomatic	10	RT-PCR	Universal	2	Untyped: 41% (261/641)
Li et al.[26]	Hubei, China	January 23 – February 25, 2020	476	Symptomatic	14	RT-PCR	Symptomatic	1	Untyped: 10.8% (110/1,020)

© 2022 Madewell ZJ et al. *JAMA Network Open.*

Authors	Location	Study period	No. index cases	Index case symptom status	Duration of follow-up (days)	Test used to diagnose contacts	Universal testing or only symptomatic	Number of tests per contact	Household SAR (infected/total)
Liu et al.[27]	Los Angeles, USA	December 2020 – February 2021	15	Symptomatic	14	RT-PCR	Universal	5	Untyped: 34.0% (17/50)
Loss et al.[28]	Germany	October 2020 – June 2021	24	Symptomatic and asymptomatic	12	RT-PCR	Universal	Multiple	Untyped: 53.3% (24/45)
Lyngse et al.[29]	Denmark	June 21, 2021 – October 26, 2021	8,262	Symptomatic and asymptomatic	14	RT-PCR and antigen tests	Universal	At least 1	Delta: 21.7% (11,631/53,584)
Lyngse et al.[30]	Denmark	December 9, 2021 – December 21, 2021	11,939	Symptomatic and asymptomatic	7	RT-PCR and antigen tests	Universal	At least 1	Delta: 21.3% (4,923/23,156)<br>Omicron: 31.2% (1,474/4,718)
Lyngse et al.[31]	Denmark	December 20, 2021 – January 11, 2022	8,541	Symptomatic and asymptomatic	7	RT-PCR and antigen tests	Universal	At least 1	Omicron: 31.8% (5,702/17,945)
Ma et al.[32]	Guangzhou, China	May 21, 2021, – June 18, 2021	69	Symptomatic and asymptomatic	14	RT-PCR	Universal	1	Delta: 9.3% (15/162)
Martinez et al.[33]	Baltimore, USA	June 11, 2020 – May 20, 2021	277	Symptomatic	14	RT-PCR	Universal	1	Untyped: 45.8% (292/638)
Martínez-Baz et al.[34]	Navarre, Spain	April – August 2021	–	Symptomatic and asymptomatic	10	RT-PCR	Universal	2	Untyped: 28.5% (4,639/16,305)
Meena et al.[35]	Madurai, Tamil Nadu, India	March – May, 2020	139	Symptomatic and asymptomatic	>7 days	RT-PCR	Universal	1	Untyped: 8.1% (42/521)

© 2022 Madewell ZJ et al. *JAMA Network Open.*

Authors	Location	Study period	No. index cases	Index case symptom status	Duration of follow-up (days)	Test used to diagnose contacts	Universal testing or only symptomatic	Number of tests per contact	Household SAR (infected/total)
Meyer et al.[36]	Germany	December 31, 2020 – April 26, 2021	14	Symptomatic and asymptomatic	14	RT-PCR	Universal	2	Untyped: 45.2% (14/31)
Miller et al.[37]	England	March 30, 2020 – November 17, 2020	181	Symptomatic	14	RT-PCR	Universal	2	Untyped: 21.1% (91/431) (PCR only)
Ministry of Health NZ[38]	New Zealand	August, 2021	–	–	–	–	–	–	Delta: 45.6% (902/1976)
Montecucco et al.[39]	Genoa, Italy	October 1, 2020 – March 31, 2021	6	Symptomatic and asymptomatic	–	RT-PCR	Universal	1	Untyped: 33.3% (17/51)
Musa et al.[40]	Bosnia and Herzegovina	August 3 – December 23, 2020	360	Symptomatic	14	RT-PCR or symptom-based diagnosis	–	–	Untyped: 15.9% (119/747)
Ng et al.[41]	Singapore	September 1, 2020 and May 31, 2021	301	Symptomatic	14	RT-PCR & whole-genome sequencing	Universal	2	Delta: 22.4% (169/753)<br>Other: 13.3% (33/248)
Ng et al.[42]	Negeri Sembilan, Malaysia	February 1, 2020 – December 31, 2020	185	Symptomatic and asymptomatic	14	RT-PCR	Universal	At least 1	Untyped: 55.0% (466/848)
Ogata et al.[43]	Tsuchiura, Japan	August 2020 – February 2021	236	Symptomatic and asymptomatic	–	RT-PCR	Universal	At least 1	Untyped: 25.2% (125/496)

© 2022 Madewell ZJ et al. *JAMA Network Open.*

Authors	Location	Study period	No. index cases	Index case symptom status	Duration of follow-up (days)	Test used to diagnose contacts	Universal testing or only symptomatic	Number of tests per contact	Household SAR (infected/total)
Rajmohan et al.[44]	Thrissur, India	January 1, 2021 – February 28, 2021	101	Symptomatic and asymptomatic	14	RT-PCR or Rapid Antigen Test	Universal	1	Untyped: 40.7% (185/387)
Ratovoson et al.[45]	Madagascar	March 19 – July 30, 2020	33	Symptomatic and asymptomatic	21	RT-PCR	Universal	4	Untyped: 38.8% (56/179)
Remón-Berrade et al.[46]	Navarre, Spain	March 2 – May 26, 2020	89	Symptomatic	–	RT-PCR or Antigen Test	Universal	1	Untyped: 14.1% (46/326)
Sachdev et al.[47]	San Francisco, USA	January 29 – July 2, 2021	105	Symptomatic and asymptomatic	14	RT-PCR, loop-mediated amplification, or antigen Test	Universal	–	Overall: 28.2% (20/71)<br>Delta: 33.3% (5/16)<br>Alpha: 25.0% (2/8)<br>Gamma: 0% (0/3)<br>Beta: 20.0% (1/5)<br>Iota: 55.6% (5/9)<br>Kappa: 0% (0/2)
Singanayagam et al.[48]	U.K.	Sept 13, 2020 – Sept 15, 2021	138	Symptomatic	14-20	RT-PCR & whole-genome sequencing	Universal	Daily	Delta: 25.9% (53/205)
Smith-Jeffcoat et al.[49]	New York City, USA	November 18 – December 20, 2021	16	Symptomatic	14	RT-PCR and antigen tests	Universal	1	Omicron: 33.3% (6/18)
Song et al.[50]	South Korea	November – December 2021	25	Symptomatic and asymptomatic	14	–	Universal	–	Omicron: 50% (18/36)
Sorioano-Arandes et al.[51]	Catalonia, Spain	July 1, 2020 – October 31, 2020	80	Symptomatic and asymptomatic	–	RT-PCR or antigen testing	Universal	–	Untyped: 64.8% (560/864)

© 2022 Madewell ZJ et al. *JAMA Network Open.*

Authors	Location	Study period	No. index cases	Index case symptom status	Duration of follow-up (days)	Test used to diagnose contacts	Universal testing or only symptomatic	Number of tests per contact	Household SAR (infected/total)
Tanaka et al.[52]	Osaka Prefecture, Japan	December 1–20, 2020 & April 20, 2021 – May 3, 2021	307	Symptomatic	14	RT-PCR	Universal	At least 1	Pre-existing virus: 19.3% (56/290)<br>Alpha: 38.7% (48/124)
ur Rehman et al.[53]	Islamabad, Pakistan	March 21 – April 4, 2020	–	Symptomatic	14	RT-PCR	Universal	1	Untyped: 36.8% (14/38)
Watanapokasin et al.[54]	Bangkok, Thailand	May 1 – June 30, 2021	30	Symptomatic	14	RT-PCR	Universal	–	Alpha: 48.6% (17/35)<br>Delta: 34.9% (15/43).
Yi et al.[55]	Jeju, South Korea	August 3 – August 10, 2021	25	Symptomatic and asymptomatic	14	RT-PCR	Universal	1	Delta: 23.9% (11/46)

© 2022 Madewell ZJ et al. *JAMA Network Open.*

eTable 3. References for Studies Included in Figure 1 of SAR Over Time

Studies	% (95% CI)	Number of studies	References
All	22.9% (20.3%-25.8%)	135	1-135
- New studies identified (between June 18, 2021 and March 8, 2022)		58	1-58
- Studies from previous review[136] (through June 17, 2021)[a]		77	59-135
Midpoints in 2021 or 2022	37.3% (32.7%-42.1%)	33	1,3,7,9-14,17,19,20,22,23,25,27-31,34,36,38,41,44,47,48,50,52,54,55,57,98
Midpoints through April 2020	15.5% (13.2%-18.2%)	63	5,18,21,24,26,35,53,63-68,70-72,74,76-79,82-86,89-91,93-97,99,100,102-107,109,110,112,114,115,117,119,121,123,125-135
Midpoints from January 2020 to June 2020	16.7% (14.3%-19.3%)	80	5,15,16,18,21,24,26,35,45,46,53,59-61,63-72,74,76-79,81-97,99-107,109,110,112-117,119,121-123,125-135
Midpoints from July 2020 to December 2020	32.2% (25.4%-39.8%)	21	2,4,6,8,27,33,39,40,42,43,51,56,58,62,80,108,111,118,120,124
Midpoints from January 2021 to June 2021	35.3% (29.9%-41.1%)	23	1,7,9-11,14,17,19,23,25,27,28,32,34,36,41,44,47,48,52,54,57,98
Midpoints > June 2021	40.6% (33.1%-48.6%)	12	3,12,13,20,22,29-31,38,49,50,55

[a]5 studies[137-141] were excluded that did not include laboratory-confirmed infections and 1[142] that included only asymptomatic index cases

© 2022 Madewell ZJ et al. *JAMA Network Open.*

eTable 4. Risk of Bias Assessment for Studies Included in Review of Household Transmissibility of SARS-CoV-2 Using the Same Modified Version of the Newcastle–Ottawa Quality Assessment Scale for Observational Studies Used by Fung et al[143]

	Selection			Comparability	Outcome				
**Author**	**Representativeness of the index cases in region (2 points)[a]**	**Index case definition (1 point)[b]**	**Sample size (1 point)[c]**	**Household SAR disaggregated by index or contact covariates (1 point)[d]**	**Universal or symptom-based testing (1 point)[e]**	**Follow-up duration (2 points)[f]**	**Number of tests per contact (1 point)[g]**	**Total points**	**Risk of bias[h]**
Baker et al.[3]	+	+	+	+	+	++	0	7	Low
Cheng et al.[7]	+	+	0	+	+	++	+	7	Low
Cohen et al.[10]	++	+	+	+	+	++	+	9	Low
De Gier et al.[11]	++	+	+	+	+	++	0	8	Low
De Gier et al.[12]	++	+	+	+	+	++	+	9	Low
Del Águila-Mejía et al.[13]	+	+	+	+	+	+	+	7	Low
Dougherty et al.[14]	+	+	0	0	+	++	0	5	Moderate
Gazit et al.[17]	++	+	+	+	+	+	0	7	Low
Gorgels et al.[19]	+	+	0	+	+	++	+	7	Low
Harris et al.[1]	++	+	+	+	+	++	+	9	Low
Hwang et al.[20]	++	+	0	0	+	++	+	7	Low
Jalali et al.[22]	++	+	+	+	+	+	+	8	Low
Kang et al.[23]	++	+	0	0	+	++	+	7	Low
Layan et al.[25]	+	+	+	+	+	+	+	7	Low
Loenenbach et al.[98]	+	+	0	+	0	++	0	5	Moderate
Lyngse et al.[29]	++	+	+	+	+	++	0	8	Low
Lyngse et al.[30]	++	+	+	+	+	+	0	7	Low
Lyngse et al.[31]	++	+	+	+	+	+	0	7	Low
Lyngse et al.[57]	++	+	+	+	+	++	0	8	Low
Ma et al.[32]	+	+	0	0	+	++	0	5	Moderate
Martínez-Baz et al.[34]	++	+	+	+	+	+	+	8	Low
Meyer et al.[36]	0	+	0	+	+	++	+	6	Moderate
Ministry of Health NZ[38]	++	0	+	0	0	0	0	3	High
Ng et al.[41]	++	+	+	+	+	++	+	9	Low
Sachdev et al.[47]	+	+	0	+	+	++	0	6	Moderate
Singanayagam et al.[48]	+	+	0	+	+	++	+	7	Low
Smith-Jeffcoat et al.[49]	0	+	0	+	+	++	0	5	Moderate

© 2022 Madewell ZJ et al. *JAMA Network Open.*

Song et al.[50]	0	+	0	+	+	+	+	5	Moderate
Tanaka et al.[52]	++	+	0	+	+	++	+	8	Low
Watanapokasin et al.[54]	0	+	0	+	+	++	0	5	Moderate
Yi et al.[55]	0	+	0	+	+	++	0	5	Moderate

[a] ++: Representative of COVID-19 cases in region; +: Somewhat representative; 0: Poorly described or not representative of cases in region
[b] +: Index case identified by date of onset of symptoms and/or test dates; 0: First case not clearly defined
[c] +: ≥300 contacts; 0: <300 contacts
[d] +: Secondary attack rate disaggregated by ≥1 covariate; 0: Secondary attack rate not disaggregated by any covariates
[e] +: Tested all contacts (both symptomatic and asymptomatic); 0: Only tested symptomatic contacts
[f] ++: ≥14 days; +: 7 days; 0: <7 days or not specified
[g] +: ≥2 tests; 0: 1 test or not described
[h] High: ≤3 points; moderate: 4–6 points; low: ≥7 points

SARs for Omicron (42.7%; 95%CI, 34.3%-51.5%) (5 studies[3,13,22,30,31]), Alpha (35.8%; 95%CI, 32.9%-38.9%) (7 studies[10,11,17,19,25,52,57]), Delta (29.1%; 95%CI, 21.7%-37.8%) (10 studies[10,12,13,20,22,23,29,30,41,48]), and Beta (22.5%; 95%CI, 18.6%-26.9%) (2 studies[7,10]) did not significantly change from those reported in Figure 2 when restricting to studies with low risk of bias.

© 2022 Madewell ZJ et al. *JAMA Network Open.*

eTable 5. Pairwise Analyses of Index Case Vaccination Status Using Only Studies in Which SARs Were Reported From Both Relevant Subgroups

Variant	Subgroup estimates: % (95%CI)	Number of study pairs	References
To all contacts regardless of vaccination status			
All	Fully vaccinated: 22.8% (15.3%-32.7%)<br>Unvaccinated: 35.5% (27.3%-44.6%)	11	3,11-13,17,22,25,29,36,41,48
All	Partially vaccinated: 26.2% (11.5%-49.2%)<br>Unvaccinated: 28.0% (17.3%-42.0%)	7	1,3,11,12,22,41,48
All	Fully vaccinated: 24.9% (14.6%-39.2%)<br>Partially vaccinated: 31.7% (15.0%-55.0%)	6	3,11,12,22,41,48
Alpha	Fully vaccinated: 10.7% (9.0%-12.8%)<br>Unvaccinated: 36.3% (31.3%-41.6%)	4	11,17,25,36
Delta	Fully vaccinated: 24.8% (17.2%-34.3%)<br>Unvaccinated: 28.3% (19.9%-38.6%)	6	12,13,22,29,41,48
Delta	Partially vaccinated: 22.5% (11.2%-40.1%)<br>Unvaccinated: 24.6% (18.1%-32.6%)	4	12,22,41,48
Delta	Fully vaccinated: 21.9% (14.4%-32.0%)<br>Partially vaccinated: 22.5% (11.2%-40.1%)	4	12,22,41,48
Omicron	Booster vaccinated: 38.0% (29.9%-46.8%)<br>Unvaccinated: 52.5% (40.2%-64.5%)	3	3,22,31
Omicron	Fully vaccinated: 50.8% (47.9%-53.8%)<br>Unvaccinated: 55.5% (48.4%-62.3%)	3	3,13,22
Omicron	Partially vaccinated: 76.8% (7.7%-99.2%)<br>Unvaccinated: 58.6% (53.9%-63.1%)	2	3,22
Omicron	Booster vaccinated: 38.0% (29.9%-46.8%)<br>Fully vaccinated: 50.8% (47.9%-53.8%)	3	3,22,31
Omicron	Booster vaccinated: 44.1% (37.6%-50.9%)<br>Partially vaccinated: 76.8% (7.7%-99.2%)	2	3,22
Omicron	Fully vaccinated: 51.0% (47.8%-54.3%)<br>Partially vaccinated: 76.8% (7.7%-99.2%)	2	3,22
To unvaccinated contacts only			
All	Fully vaccinated: 12.0% (10.0%-14.2%)<br>Unvaccinated: 30.9% (23.9%-38.8%)	4	11,12,17,48
All	Partially vaccinated: 14.8% (6.7%-29.4%)<br>Unvaccinated: 19.5% (11.1%-31.8%)	3	1,11,12

© 2022 Madewell ZJ et al. *JAMA Network Open.*

All	Fully vaccinated: 11.7% (9.8%-13.9%)<br>Partially vaccinated: 23.9% (16.7%-32.9%)	2	[11,12]

© 2022 Madewell ZJ et al. *JAMA Network Open.*

eTable 6. Pairwise Analyses of Household Contact Vaccination Status Using Only Studies in Which SARs Were Reported From Both Relevant Subgroups

Variant	Subgroup estimates: % (95%CI)	Number of study pairs	References
**From all index cases regardless of vaccination status**			
All	Fully vaccinated: 18.8% (12.6%-27.1%)<br>Unvaccinated: 36.5% (30.5%-43.0%)	12	3,11,12,17,22,25,29,34,41,47,48,55
All	Partially vaccinated: 27.8% (20.0%-37.1%)<br>Unvaccinated: 39.6% (32.3%-47.4%)	8	3,12,22,34,41,47,48,55
All	Fully vaccinated: 23.9% (14.7%-36.4%)<br>Partially vaccinated: 27.8% (20.0%-37.1%)	8	3,12,22,34,41,47,48,55
Alpha	Fully vaccinated: 10.5% (7.9%-13.8%)<br>Unvaccinated: 38.4% (34.4%-42.5%)	3	11,17,25
Delta	Booster vaccinated: 11.3% (9.8%-13.0%)<br>Unvaccinated: 36.1% (24.2%-50.0%)	2	22,30
Delta	Fully vaccinated: 17.1% (11.6%-24.6%)<br>Unvaccinated: 30.1% (23.2%-38.1%)	6	12,22,29,41,48,55
Delta	Partially vaccinated: 23.6% (17.2%-31.6%)<br>Unvaccinated: 34.7% (25.5%-45.1%)	4	22,41,48,55
Delta	Fully vaccinated: 21.0% (12.8%-32.5%)<br>Partially vaccinated: 23.6% (17.2%-31.6%)	4	22,41,48,55
Omicron	Booster vaccinated: 32.7% (24.5%-42.2%)<br>Unvaccinated: 43.9% (32.2%-56.2%)	4	3,22,30,31
Omicron	Fully vaccinated: 51.2% (47.8%-54.7%)<br>Unvaccinated: 57.4% (52.8%-61.9%)	2	3,22
Omicron	Partially vaccinated: 56.6% (48.2%-64.7%)<br>Unvaccinated: 57.4% (52.8%-61.9%)	2	3,22
Omicron	Booster vaccinated: 41.7% (34.9%-48.9%)<br>Fully vaccinated: 51.2% (47.8%-54.7%)	2	3,22
Omicron	Booster vaccinated: 41.7% (34.9%-48.9%)<br>Partially vaccinated: 56.6% (48.2%-64.7%)	2	3,22
Omicron	Fully vaccinated: 51.2% (47.8%-54.7%)<br>Partially vaccinated: 56.6% (48.2%-64.7%)	2	3,22
**From unvaccinated index cases only**			
All	Fully vaccinated: 11.0% (10.1%-12.0%)<br>Unvaccinated: 32.% (24.7%-40.6%)	4	11,12,17,48

© 2022 Madewell ZJ et al. *JAMA Network Open.*

All	Partially vaccinated: 28.5% (26.3%-30.8%)<br>Unvaccinated: 35.2% (34.9%-35.5%)	2	[11,48]
All	Fully vaccinated: 11.2% (10.0%-12.5%)<br>Partially vaccinated: 28.5% (26.3%-30.8%)	2	[11,48]

© 2022 Madewell ZJ et al. *JAMA Network Open.*

eTable 7. Household Secondary Attack Rates by Vaccine Type and Contact Vaccination Status With All Index Cases Included Regardless of Vaccination Status

Vaccine	SAR (95% CI)	Number of studies	References
Full vaccination			
Ad26.COV2.S	34.2% (14.4%-61.5%)	4	[12,29,34,47]
BNT162b2	15.2% (14.6%-16.0%)	4	[12,29,34,47]
ChAdOx1-S	13.6% (7.4%-23.7%)	3	[12,29,34]
mRNA-1273	9.5% (8.6%-10.6%)	4	[12,29,34,47]
Partial vaccination			
BNT162b2	30.5% (18.5%-45.9%)	2	[12,34]
ChAdOx1-S	29.5% (24.0%-35.7%)	2	[12,34]
mRNA-1273	17.5% (13.7%-22.3%)	2	[12,34]
Unvaccinated	35.4% (29.0%-42.5%)	4	[12,29,34,47]

© 2022 Madewell ZJ et al. *JAMA Network Open.*

eTable 8. Vaccination Status Definitions for Studies That Reported Household Secondary Attack Rates by Vaccination Status of Index Cases or Contacts

Study	Vaccination definitions
Baker et al.[3]	Received a booster dose was defined as having received an additional dose after completion of the primary COVID-19 vaccination series before the index date. Fully vaccinated was defined as completion of the primary vaccination series ≥2 weeks before the index date and stratified into completion <5 months or ≥5 months before the index date. Some persons who were fully vaccinated had unknown dates for completion of their primary vaccination series. Partially vaccinated was defined as having only 1 dose of a 2-dose series or completing the primary vaccination series <2 weeks before the index date.
De Gier et al.[11]	Partly vaccinated was defined as having received the first dose of a two-dose schedule at least 14 days before onset of symptoms. Fully vaccinated was defined as having completed a two-dose schedule at least 7 days or the one-dose Janssen schedule at least 14 days before symptom onset.
De Gier et al.[12]	Full vaccination defined as 14 or more days after the second-dose of Comirnaty (BNT162b2 mRNA; BioNTech-Pfizer, Mainz, Germany/New York, United States), Spikevax (mRNA-1273, Moderna, Cambridge, United States) or Vaxzevria (ChAdOx1 nCoV-19; Oxford-AstraZeneca, Cambridge, United Kingdom) or 28 or more days after one-dose of Janssen COVID-19 vaccine (Ad26.COV2-S, Janssen-Cilag International NV, Beerse, Belgium).
Del Águila-Mejía et al.[13]	Vaccination status was dichotomized to either non vaccinated or fully vaccinated as per each vaccine's protocol.
Harris et al.[1]	Vaccinated index cases defined as having been vaccinated 21 days or more prior to testing positive for COVID-19 based on evidence of the time needed for the vaccine to provide a sufficient level of immunity. Non-vaccinated index cases were defined as not having received a vaccine prior to testing positive. Households where the index case received the vaccine less than 21 days before testing positive were excluded from this analysis. Most of the vaccinated index patients (93%) had received only the first dose of vaccine.
Gazit et al.[17]	Participants were classified into one of three vaccination-status groups at the time of the index case (the confirmed exposure): Unvaccinated; Recently Vaccinated Once, i.e. those vaccinated with the first vaccine dose within 0-7 days before the index infection, and Fully Vaccinated, i.e. those who were 7 or more days post the second dose by the time of the confirmed exposure.
Jalali et al.[22]	To define the vaccine status of the household contacts, they used the test date of the primary case and compared it with the contacts vaccination dates: 1.Unvaccinated: A contact was considered unvaccinated if the primary case's test date is before the contact's first dose. 2.Partially vaccinated: A contact was considered partially vaccinated if he/she had received 1 dose of vaccine (mRNA Vaccines or AstraZeneca vaccine) prior to the test date of his/her primary case. Contacts who had received dose 2 within the last week before the primary case's test date were also considered partly vaccinated. 3.Fully vaccinated: A contact was considered fully vaccinated if he/she had received dose 2 (mRNA) at least 1 week prior to the test date of his/her primary case. 4.Booster vaccinated: A contact was considered booster vaccinated if he/she had received dose 3 at least 1 week prior to the test date of his/her primary case. The time interval between the second and the third doses should be >= 120 days. The vaccine status of the primary cases was defined based on their test date and their vaccination dates. Individuals with J&J vaccine were excluded from the study. We excluded households where two individuals tested positive on the same day to ensure a unique index case in each household.

© 2022 Madewell ZJ et al. *JAMA Network Open.*

Layan et al.[25]	Cases were considered vaccinated if their infection occurred >7 days after the 2nd dose. Similarly, household contacts were considered vaccinated if their exposure to the index case occurred >7 days after the 2nd dose
Lyngse et al.[29]	Individuals who had not received a first dose were classified as not vaccinated. The definitions of full vaccinations were: Comirnaty (Pfizer/BioNTech): 7 days after second dose; Vaxzevria (AstraZeneca): 15 days after second dose; Spikevax (Moderna): 14 days after second dose; COVID-19 vaccine Janssen (Johnson & Johnson): 14 days after vaccination. If an individual was cross vaccinated (mainly first dose of Vaxzevria and second dose of Comirnaty), the definition of the second dose vaccination was used. Individuals that were in the period between the first dose and fully vaccinated were defined as partially vaccinated and excluded. Any individual that had received a booster vaccination was also excluded. Lastly, all households with a previous infection (positive RT-PCR test) were excluded.
Lyngse et al.[30]	Individuals were classified by vaccination status into three groups: i) unvaccinated; ii) fully vaccinated (defined by the vaccine used, Comirnaty (Pfizer/BioNTech): 7 days after second dose; Vaxzevria (AstraZeneca): 15 days after second dose; Spikevax (Moderna): 14 days after second dose; Janssen (Johnson & Johnson): 14 days after vaccination, and 14 days after the second dose for cross vaccinated individuals) or 14 days after previous infection; or iii) booster-vaccinated (defined by 7 days after the booster vaccination. Partially vaccinated individuals were regarded as unvaccinated in this study.
Lyngse et al.[31]	The vaccination status of all individuals was classified into three groups: i) unvaccinated (including partially vaccinated individuals); ii) fully vac92 cinated (defined by the vaccine used, Comirnaty (Pfizer/BioNTech): 7 days after second dose; Vaxzevria (AstraZeneca): 15 days after second dose; Spikevax (Moderna): 14 days after second dose; Janssen (Johnson & Johnson): 14 days after vaccination, and 14 days after the second dose for cross vaccinated individuals) or 14 days after previous infection; or iii) booster-vaccinated, defined by 7 days after the booster vaccination.
Martínez-Baz et al.[34]	A person was considered fully vaccinated ≥ 14 days after receiving one dose of Janssen or the second dose of other vaccines, and partially vaccinated ≥ 14 days after receiving only the first dose of Spikevax, Comirnaty or Vaxzevria.
Meyer et al.[36]	Not defined, but the two secondary cases found among household contacts of vaccinated index cases were diagnosed 25 days after the second vaccination.
Ng et al.[41]	Both index cases and close contacts were considered partially vaccinated if they had received one vaccine dose before the day the quarantine order was issued, or were within 14 days of the second dose on the day the quarantine order was issued. If more than 14 days had elapsed after their second dose, they were taken to be fully vaccinated.
Sachdev et al.[47]	Partially vaccinated patients were defined as patients who received at least 1 dose of vaccine but were not fully vaccinated. Fully vaccinated patients were defined as patients who had received a second mRNA vaccine dose or a single-dose viral vector vaccine ≥14 days from symptom onset or collection of a positive specimen
Singanayagam et al.[48]	Participant defined as unvaccinated if they had not received a single dose of a COVID-19 vaccine at least 7 days before enrolment, partially vaccinated if they had received one vaccine dose at least 7 days before study enrolment, and fully vaccinated if they had received two doses of a COVID-19 vaccine at least 7 days before study enrolment.

© 2022 Madewell ZJ et al. *JAMA Network Open.*

eReferences

1. Harris RJ, Hall JA, Zaidi A, Andrews NJ, Dunbar JK, Dabrera G. Effect of Vaccination on Household Transmission of SARS-CoV-2 in England. *New England Journal of Medicine*. 2021;
2. Afonso ET, Marques SM, Costa LD, et al. Secondary household transmission of SARS-CoV-2 among children and adolescents: Clinical and epidemiological aspects. *Pediatric Pulmonology*. 2021;
3. Baker JM. SARS-CoV-2 B. 1.1. 529 (Omicron) Variant Transmission Within Households—Four US Jurisdictions, November 2021–February 2022. *MMWR Morbidity and Mortality Weekly Report*. 2022;71
4. Bistaraki A, Roussos S, Tsiodras S, Sypsa V. Age-dependent effects on infectivity and susceptibility to SARS-CoV-2 infection: results from nationwide contact tracing data in Greece. *Infectious Diseases*. 2021:1-10.
5. Burke RM, Calderwood L, Killerby ME, et al. Patterns of Virus Exposure and Presumed Household Transmission among Persons with Coronavirus Disease, United States, January–April 2020. *Emerging Infectious Diseases*. 2021;27(9):2323.
6. Calvani M, Cantiello G, Cavani M, et al. Reasons for SARS-CoV-2 infection in children and their role in the transmission of infection according to age: a case-control study. *Ital J Pediatr*. Sep 27 2021;47(1):193. doi:10.1186/s13052-021-01141-1
7. Cheng VC-C, Siu GK-H, Wong S-C, et al. Complementation of contact tracing by mass testing for successful containment of beta COVID-19 variant (SARS-CoV-2 VOC B. 1.351) epidemic in Hong Kong. *The Lancet Regional Health-Western Pacific*. 2021;17:100281.
8. Chu VT, Yousaf AR, Chang K, et al. Household transmission of SARS-CoV-2 from children and adolescents. *New England Journal of Medicine*. 2021;385(10):954-956.
9. Clifford S, Waight P, Hackman J, et al. Effectiveness of BNT162b2 and ChAdOx1 against SARS-CoV-2 household transmission: a prospective cohort study in England. *medRxiv*. 2021:2021.11.24.21266401. doi:10.1101/2021.11.24.21266401
10. Cohen C, Kleynhans J, von Gottberg A, et al. SARS-CoV-2 incidence, transmission and reinfection in a rural and an urban setting: results of the PHIRST-C cohort study, South Africa, 2020-2021. *medRxiv*. 2021:2021.07.20.21260855. doi:10.1101/2021.07.20.21260855
11. de Gier B, Andeweg S, Joosten R, et al. Vaccine effectiveness against SARS-CoV-2 transmission and infections among household and other close contacts of confirmed cases, the Netherlands, February to May 2021. *Eurosurveillance*. 2021;26(31):2100640.
12. de Gier B, Andeweg S, Backer JA, et al. Vaccine effectiveness against SARS-CoV-2 transmission to household contacts during dominance of Delta variant (B.1.617.2), the Netherlands, August to September 2021. *Eurosurveillance*. 2021;26(44):2100977. doi:doi:https://doi.org/10.2807/1560-7917.ES.2021.26.44.2100977
13. Águila-Mejía JD, Wallmann R, Calvo-Montes J, Rodríguez-Lozano J, Valle-Madrazo T, Aginagalde-Llorente A. Secondary Attack Rates, Transmission, Incubation and Serial Interval Periods of first SARS-CoV-2 Omicron variant cases in a northern region of Spain. *Research Square*. 2022/01/24 2022;doi:10.21203/rs.3.rs-1279005/v1
14. Dougherty K, Mannell M, Naqvi O, Matson D, Stone J. SARS-CoV-2 B. 1.617. 2 (Delta) variant COVID-19 outbreak associated with a gymnastics facility—Oklahoma, April–May 2021. *Morbidity and Mortality Weekly Report*. 2021;70(28):1004.
15. Dub T, Nohynek H, Hagberg L, et al. High secondary attack rate and persistence of SARS-CoV-2 antibodies in household transmission study participants, Finland 2020. 2021;
16. Friedman-Klabanoff DJ, Fitzpatrick MC, Deming ME, et al. Risk of Severe Acute Respiratory Syndrome Coronavirus 2 Acquisition Is Associated With Individual Exposure but Not Community-Level Transmission. *The Journal of Infectious Diseases*. 2022;doi:10.1093/infdis/jiac029
17. Gazit S, Mizrahi B, Kalkstein N, et al. BNT162b2 mRNA Vaccine Effectiveness Given Confirmed Exposure: Analysis of Household Members of COVID-19 Patients. *Clinical Infectious Diseases*. 2021;doi:10.1093/cid/ciab973
18. Ge Y, Martinez L, Sun S, et al. COVID-19 transmission dynamics among close contacts of index patients with COVID-19: a population-based cohort study in Zhejiang province, China. *JAMA Internal Medicine*. 2021;181(10):1343-1350.

© 2022 Madewell ZJ et al. *JAMA Network Open.*

19. Gorgels K, Alphen L, Hackert BvdVV, et al. Increased Transmissibility of SARS-CoV-2 Alpha Variant (B.1.1.7) in Children: Three Large Primary School Outbreaks Revealed by Whole Genome Sequencing in the Netherlands. *Research Square*. 2021/12/10 2021;doi:10.21203/rs.3.rs-1107495/v1
20. Hwang H, Lim J-S, Song S-A, et al. Transmission dynamics of the Delta variant of SARS-CoV-2 infections in South Korea. *The Journal of Infectious Diseases*. 2021;doi:10.1093/infdis/jiab586
21. Jagdale GR, Parande MA, Borle P, et al. Secondary Attack Rate among the Contacts of COVID-19 Patients at the Beginning of the Pandemic in Pune City of Western Maharashtra, India. *Journal of Communicable Diseases (E-ISSN: 2581-351X & P-ISSN: 0019-5138)*. 2021;53(3):51-59.
22. Jalali N, Brustad HK, Frigessi A, et al. Increased household transmission and immune escape of the SARS-CoV-2 Omicron variant compared to the Delta variant: evidence from Norwegian contact tracing and vaccination data. *medRxiv*. 2022:2022.02.07.22270437. doi:10.1101/2022.02.07.22270437
23. Kang M, Xin H, Yuan J, et al. Transmission dynamics and epidemiological characteristics of Delta variant infections in China. *Medrxiv*. 2021;
24. Karumanagoundar K, Raju M, Ponnaiah M, et al. Secondary attack rate of COVID-19 among contacts and risk factors, Tamil Nadu, March–May 2020: a retrospective cohort study. *BMJ open*. 2021;11(11):e051491.
25. Layan M, Gilboa M, Gonen T, et al. Impact of BNT162b2 vaccination and isolation on SARS-CoV-2 transmission in Israeli households: an observational study. *MedRxiv*. 2021;
26. Li Y, Liu J, Yang Z, et al. Transmission of Severe Acute Respiratory Syndrome Coronavirus 2 to Close Contacts, China, January–February 2020. *Emerging Infectious Diseases*. 2021;27(9):2288.
27. Liu PY, Gragnani CM, Timmerman J, et al. Pediatric Household Transmission of Severe Acute Respiratory Coronavirus-2 Infection—Los Angeles County, December 2020 to February 2021. *The Pediatric Infectious Disease Journal*. 2021;40(10):e379.
28. Loss J, Wurm J, Varnaccia G, et al. Transmission of SARS-CoV-2 among children and staff in German daycare centers: results from the COALA study. *medRxiv*. 2021:2021.12.21.21268157. doi:10.1101/2021.12.21.21268157
29. Lyngse FP, Mølbak K, Denwood M, et al. Effect of Vaccination on Household Transmission of SARS-CoV-2 Delta VOC. *medRxiv*. 2022:2022.01.06.22268841. doi:10.1101/2022.01.06.22268841
30. Lyngse FP, Mortensen LH, Denwood MJ, et al. SARS-CoV-2 Omicron VOC Transmission in Danish Households. *medRxiv*. 2021:2021.12.27.21268278. doi:10.1101/2021.12.27.21268278
31. Lyngse FP, Kirkeby CT, Denwood M, et al. Transmission of SARS-CoV-2 Omicron VOC subvariants BA.1 and BA.2: Evidence from Danish Households. *medRxiv*. 2022:2022.01.28.22270044. doi:10.1101/2022.01.28.22270044
32. Ma X, Wu K, Li Y, et al. Contact tracing period and epidemiological characteristics of an outbreak of the SARS-CoV-2 Delta variant in Guangzhou. *International Journal of Infectious Diseases*. 2022/04/01/ 2022;117:18-23. doi:https://doi.org/10.1016/j.ijid.2022.01.034
33. Martinez DA, Klein EY, Parent C, et al. Latino Household Transmission of Severe Acute Respiratory Syndrome Coronavirus 2. *Clinical Infectious Diseases*. 2021;
34. Martínez-Baz I, Trobajo-Sanmartín C, Miqueleiz A, et al. Product-specific COVID-19 vaccine effectiveness against secondary infection in close contacts, Navarre, Spain, April to August 2021. *Eurosurveillance*. 2021;26(39):2100894.
35. Meena MS, Priya S, Thirukumaran R, Gowrilakshmi M, Essakiraja K, Madhumitha M. Factors influencing the acquisition of COVID infection among high-risk contacts of COVID-19 patients in Madurai district-A case control study. *Journal of Family Medicine and Primary Care*. 2022;11(1):182-189.
36. Meyer ED, Sandfort M, Bender J, et al. Two doses of the mRNA BNT162b2 vaccine reduce severe outcomes, viral load and secondary attack rate: evidence from a SARS-CoV-2 Alpha outbreak in a nursing home in Germany, January-March 2021. *medRxiv*. 2021:2021.09.13.21262519. doi:10.1101/2021.09.13.21262519

© 2022 Madewell ZJ et al. *JAMA Network Open.*

37. Miller E, Waight PA, Andrews NJ, et al. Transmission of SARS-CoV-2 in the household setting: A prospective cohort study in children and adults in England. *Journal of Infection*. 2021;83(4):483-489.
38. Ministry of Health NZ. COVID-19 Variants Update. December 3, 2021. https://www.health.govt.nz/system/files/documents/pages/22-november-2021-variants-update-summary.pdf
39. Montecucco A, Dini G, Rahmani A, et al. Investigating SARS-CoV-2 transmission among co-workers in a University of Northern Italy during COVID-19 pandemic: an observational study. *La Medicina del Lavoro | Work, Environment and Health*. 12/23 2021;112(6):429-435. doi:10.23749/mdl.v112i6.12527
40. Musa S, Kissling E, Valenciano M, et al. Household transmission of SARS-CoV-2: a prospective observational study in Bosnia and Herzegovina, August–December 2020. *International Journal of Infectious Diseases*. 2021;112:352-361.
41. Ng OT, Koh V, Chiew CJ, et al. Impact of Delta Variant and Vaccination on SARS-CoV-2 Secondary Attack Rate Among Household Close Contacts. *The Lancet Regional Health-Western Pacific*. 2021;17:100299.
42. Ng DCE, Tan KK, Chin L, et al. Risk factors associated with household transmission of SARS-CoV-2 in Negeri Sembilan, Malaysia. *Journal of paediatrics and child health*. 2021;
43. Ogata T, Irie F, Ogawa E, et al. Secondary Attack Rate among Non-Spousal Household Contacts of Coronavirus Disease 2019 in Tsuchiura, Japan, August 2020–February 2021. *International Journal of Environmental Research and Public Health*. 2021;18(17):8921.
44. Rajmohan P, Jose P, Thodi JBA, et al. Dynamics of transmission of COVID-19 cases and household contacts: A prospective cohort study. *Journal of Acute Disease*. 2021;10(4):162.
45. Ratovoson R, Razafimahatratra R, Randriamanantsoa L, et al. Household transmission of COVID-19 among the earliest cases in Antananarivo, Madagascar. *Influenza Other Respir Viruses*. Aug 10 2021;doi:10.1111/irv.12896
46. Remón-Berrade M, Guillen-Aguinaga S, Sarrate-Adot I, et al. Risk of Secondary Household Transmission of COVID-19 from Health Care Workers in a Hospital in Spain. *Epidemiologia*. 2022;3(1):1-10.
47. Sachdev DD, Chew Ng R, Sankaran M, et al. Contact tracing outcomes among household contacts of fully vaccinated COVID-19 patients - San Francisco, California, January 29-July 2, 2021. *Clin Infect Dis*. Dec 20 2021;doi:10.1093/cid/ciab1042
48. Singanayagam A, Hakki S, Dunning J, et al. Community transmission and viral load kinetics of the SARS-CoV-2 delta (B. 1.617. 2) variant in vaccinated and unvaccinated individuals in the UK: a prospective, longitudinal, cohort study. *The Lancet Infectious Diseases*. 2021;
49. Smith-Jeffcoat SE. Multistate outbreak of SARS-CoV-2 B. 1.1. 529 (Omicron) variant infections among persons in a social network attending a convention—New York City, November 18–December 20, 2021. *MMWR Morbidity and Mortality Weekly Report*. 2022;71
50. Song JS, Lee J, Kim M, et al. Serial Intervals and Household Transmission of SARS-CoV-2 Omicron Variant, South Korea, 2021. *Emerg Infect Dis*. Feb 2 2022;28(3)doi:10.3201/eid2803.212607
51. Soriano-Arandes A, Gatell A, Serrano P, et al. Household Severe Acute Respiratory Syndrome Coronavirus 2 Transmission and Children: A Network Prospective Study. *Clin Infect Dis*. Sep 15 2021;73(6):e1261-e1269. doi:10.1093/cid/ciab228
52. Tanaka H, Hirayama A, Nagai H, et al. Increased transmissibility of the SARS-CoV-2 alpha variant in a japanese population. *International Journal of Environmental Research and Public Health*. 2021;18(15):7752.
53. ur Rehman S, Qaisrani M, Abbasi S, et al. COVID-19 outbreak in Islamabad resulting from a travel-associated primary case: A case series. *Global Biosecurity*. 2021;3(1)
54. Watanapokasin N, Siripongboonsitti T, Ungtrakul T, et al. Transmissibility of SARS-CoV-2 variants as a secondary attack in Thai households: a retrospective study. *IJID Regions*. 2021;1:1-2.
55. Yi S, Kim JM, Choe YJ, et al. SARS-CoV-2 Delta Variant Breakthrough Infection and Onward Secondary Transmission in Household. *J Korean Med Sci*. 1/ 2022;37(1):0.

56. Cerami C, Popkin-Hall ZR, Rapp T, et al. Household transmission of SARS-CoV-2 in the United States: living density, viral load, and disproportionate impact on communities of color. *Clin Infect Dis*. Aug 12 2021;doi:10.1093/cid/ciab701
57. Lyngse FP, Mølbak K, Skov RL, et al. Increased transmissibility of SARS-CoV-2 lineage B.1.1.7 by age and viral load. *Nature Communications*. 2021/12/13 2021;12(1):7251. doi:10.1038/s41467-021-27202-x
58. Tanaka ML, Marentes Ruiz CJ, Malhotra S, et al. SARS-CoV-2 Transmission Dynamics in Households With Children, Los Angeles, California. Original Research. *Frontiers in Pediatrics*. 2022-January-05 2022;9(1520)doi:10.3389/fped.2021.752993
59. Adamik B, Bawiec M, Bezborodov V, et al. Bounds on the total number of SARS-CoV-2 infections: The link between severeness rate, household attack rate and the number of undetected cases. 2020;
60. Akaishi T, Kushimoto S, Katori Y, et al. COVID-19 transmission in group living environments and households. *Scientific Reports*. 2021/06/02 2021;11(1):11616. doi:10.1038/s41598-021-91220-4
61. Areekal B, Vijayan S, Suseela MS, et al. Risk Factors, Epidemiological and Clinical Outcome of Close Contacts of COVID-19 Cases in a Tertiary Hospital in Southern India. *Journal of Clinical & Diagnostic Research*. 2021;15(3)
62. Awang H, Yaacob EL, Syed Aluawi SN, et al. A case–control study of determinants for COVID-19 infection based on contact tracing in Dungun district, Terengganu state of Malaysia. *Infectious Diseases*. 2021;53(3):222-225.
63. Bae S, Kim H, Jung T-Y, et al. Epidemiological Characteristics of COVID-19 Outbreak at Fitness Centers in Cheonan, Korea. *J Korean Med Sci*. 8/ 2020;35(31)
64. Bender JK, Brandl M, Höhle M, Buchholz U, Zeitlmann N. Analysis of asymptomatic and presymptomatic transmission in SARS-CoV-2 outbreak, Germany, 2020. *Emerging infectious diseases*. 2021;27(4):1159.
65. Bi Q, Wu Y, Mei S, et al. Epidemiology and transmission of COVID-19 in 391 cases and 1286 of their close contacts in Shenzhen, China: a retrospective cohort study. *The Lancet Infectious Diseases*. 2020;
66. Böhmer MM, Buchholz U, Corman VM, et al. Investigation of a COVID-19 outbreak in Germany resulting from a single travel-associated primary case: a case series. *The Lancet Infectious Diseases*. 2020;
67. Boscolo-Rizzo P, Borsetto D, Spinato G, et al. New onset of loss of smell or taste in household contacts of home-isolated SARS-CoV-2-positive subjects. *European Archives of Oto-rhino-laryngology*. May 24 2020:1-4. doi:10.1007/s00405-020-06066-9
68. Burke RM. Active monitoring of persons exposed to patients with confirmed COVID-19—United States, January–February 2020. *MMWR Morbidity and mortality weekly report*. 2020;69
69. Charbonnier L, Rouprêt-Serzec J, Caseris M, et al. Contribution of Serological Rapid Diagnostic Tests to the Strategy of Contact Tracing in Households Following SARS-CoV-2 Infection Diagnosis in Children. Brief Research Report. *Frontiers in Pediatrics*. 2021-May-10 2021;9(217)doi:10.3389/fped.2021.638502
70. Chaw L, Koh WC, Jamaludin SA, Naing L, Alikhan MF, Wong J. Analysis of SARS-CoV-2 transmission in different settings, Brunei. *Emerging infectious diseases*. 2020;26(11):2598.
71. Chen Y, Wang AH, Yi B, et al. [Epidemiological characteristics of infection in COVID-19 close contacts in Ningbo city]. *Zhonghua Liu Xing Bing Xue Za Zhi*. May 10 2020;41(5):667-671. doi:10.3760/cma.j.cn112338-20200304-00251
72. Cheng HY, Jian SW, Liu DP, Ng TC, Huang WT, Lin HH. Contact Tracing Assessment of COVID-19 Transmission Dynamics in Taiwan and Risk at Different Exposure Periods Before and After Symptom Onset. *JAMA Intern Med*. May 1 2020;doi:10.1001/jamainternmed.2020.2020
73. Dattner I, Goldberg Y, Katriel G, et al. The role of children in the spread of COVID-19: Using household data from Bnei Brak, Israel, to estimate the relative susceptibility and infectivity of children. *PLoS computational biology*. 2021;17(2):e1008559.
74. Dawson P, Rabold EM, Laws RL, et al. Loss of Taste and Smell as Distinguishing Symptoms of COVID-19. *Clin Infect Dis*. Jun 21 2020;doi:10.1093/cid/ciaa799

© 2022 Madewell ZJ et al. *JAMA Network Open.*

75. Demko ZO, Antar AAR, Blair PW, et al. Clustering of SARS-CoV-2 infections in households of patients diagnosed in the outpatient setting in Baltimore, MD. *Open Forum Infectious Diseases*. 2021;doi:10.1093/ofid/ofab121
76. Dong XC, Li JM, Bai JY, et al. [Epidemiological characteristics of confirmed COVID-19 cases in Tianjin]. *Zhonghua Liu Xing Bing Xue Za Zhi*. May 10 2020;41(5):638-641. doi:10.3760/cma.j.cn112338-20200221-00146
77. Doung-ngern P, Suphanchaimat R, Panjagampatthana A, et al. Case-Control Study of Use of Personal Protective Measures and Risk for Severe Acute Respiratory Syndrome Coronavirus 2 Infection, Thailand. *Emerging Infectious Diseases*. 2020;26(11)
78. Draper AD, Dempsey KE, Boyd RH, et al. The first 2 months of COVID-19 contact tracing in the Northern Territory of Australia, March-April 2020. *Communicable Diseases Intelligence*. Jul 2 2020;44doi:10.33321/cdi.2020.44.53
79. Fateh-Moghadam P, Battisti L, Molinaro S, et al. Contact tracing during Phase I of the COVID-19 pandemic in the Province of Trento, Italy: key findings and recommendations. *medRxiv*. 2020;
80. Gomaa MR, El Rifay AS, Shehata M, et al. Incidence, household transmission, and neutralizing antibody seroprevalence of Coronavirus Disease 2019 in Egypt: Results of a community-based cohort. *PLOS Pathogens*. 2021;17(3):e1009413. doi:10.1371/journal.ppat.1009413
81. Grijalva CG, Rolfes MA, Zhu Y, et al. Transmission of SARS-COV-2 infections in households—Tennessee and Wisconsin, April–September 2020. *Morbidity and Mortality Weekly Report*. 2020;69(44):1631.
82. Han T. Outbreak investigation: transmission of COVID-19 starting from a spa facility in a local community in Korea. *Epidemiol Health*. 2020;0(0):e2020056-0. doi:10.4178/epih.e2020056
83. Hsu C-Y, Wang J-T, Huang K-C, Chiao-Hsin Fan A, Yeh Y-P, Li-Sheng Chen S. Household Transmission but without the Community-acquired Outbreak of COVID-19 in Taiwan. *Journal of the Formosan Medical Association*. 2021/05/03/ 2021;doi:https://doi.org/10.1016/j.jfma.2021.04.021
84. Hu P, Ma M, Jing Q, et al. Retrospective study identifies infection related risk factors in close contacts during COVID-19 epidemic. *International Journal of Infectious Diseases*. 2021;103:395-401.
85. Hu S, Wang W, Wang Y, et al. Infectivity, susceptibility, and risk factors associated with SARS-CoV-2 transmission under intensive contact tracing in Hunan, China. *Nature Communications*. 2021/03/09 2021;12(1):1533. doi:10.1038/s41467-021-21710-6
86. Hua CZ, Miao ZP, Zheng JS, et al. Epidemiological features and viral shedding in children with SARS-CoV-2 infection. *Journal of Medical Virology*. Jun 15 2020;doi:10.1002/jmv.26180
87. Islam SS, Noman ASM. Transmission Dynamics and Contact Tracing Assessment of COVID-19 in Chattogram, Bangladesh and Potential Risk of Close Contacts at Different Exposure Settings. *Bangladesh and Potential Risk of Close Contacts at Different Exposure Settings*.
88. Jashaninejad R, Doosti-Irani A, Karami M, Keramat F, Mirzaei M. Transmission of COVID-19 and its Determinants among Close Contacts of COVID-19 Patients Running title. *Journal of Research in Health Sciences*. 2021;
89. Jing Q-L, Liu M-J, Yuan J, et al. Household secondary attack rate of COVID-19 and associated determinants in Guangzhou, China: a retrospective cohort study. *The Lancet Infectious Diseases*. 2020;
90. Kim J, Choe YJ, Lee J, et al. Role of children in household transmission of COVID-19. *Archives of Disease in Childhood*. 2020:archdischild-2020-319910. doi:10.1136/archdischild-2020-319910
91. Covid-19 National Emergency Response Center Epidemiology Case Management Team Korea Centers for Disease Control Prevention. Coronavirus Disease-19: Summary of 2,370 Contact Investigations of the First 30 Cases in the Republic of Korea. *Osong Public Health Res Perspect*. 2020;11(2):81-84. doi:10.24171/j.phrp.2020.11.2.04
92. Koureas M, Speletas M, Bogogiannidou Z, et al. Transmission Dynamics of SARS-CoV-2 during an Outbreak in a Roma Community in Thessaly, Greece—Control Measures and Lessons Learned. *International Journal of Environmental Research and Public Health*. 2021;18(6):2878.

© 2022 Madewell ZJ et al. *JAMA Network Open.*

93. Kuba Y, Shingaki A, Nidaira M, et al. The characteristics of household transmission during COVID-19 outbreak in Okinawa, Japan from February to May 2020. *Japanese Journal of Infectious Diseases*. 2021:JJID. 2020.943.
94. Laxminarayan R, Wahl B, Dudala SR, et al. Epidemiology and transmission dynamics of COVID-19 in two Indian states. *Science*. Sep 30 2020;
95. Lewis NM, Chu VT, Ye D, et al. Household transmission of SARS-CoV-2 in the United States. *Clinical Infectious Diseases*. 2020;
96. Li F, Li Y-Y, Liu M-J, et al. Household transmission of SARS-CoV-2 and risk factors for susceptibility and infectivity in Wuhan: a retrospective observational study. *The Lancet Infectious Diseases*. 2021;
97. Liu T, Liang W, Zhong H, et al. Risk factors associated with COVID-19 infection: a retrospective cohort study based on contacts tracing. *Emerging Microbes & Infections*. Jul 1 2020:1-31. doi:10.1080/22221751.2020.1787799
98. Loenenbach A, Markus I, Lehfeld A-S, et al. SARS-CoV-2 variant B. 1.1. 7 susceptibility and infectiousness of children and adults deduced from investigations of childcare centre outbreaks, Germany, 2021. *Eurosurveillance*. 2021;26(21):2100433.
99. Lopez Bernal J, Panagiotopoulos N, Byers C, et al. Transmission dynamics of COVID-19 in household and community settings in the United Kingdom. *medRxiv*. 2020:2020.08.19.20177188. doi:10.1101/2020.08.19.20177188
100. Luo L, Liu D, Liao X, et al. Contact Settings and Risk for Transmission in 3410 Close Contacts of Patients With COVID-19 in Guangzhou, China: A Prospective Cohort Study. *Annals of internal medicine*. Aug 13 2020;doi:10.7326/m20-2671
101. Lyngse FP, Kirkeby CT, Halasa T, et al. COVID-19 Transmission Within Danish Households: A Nationwide Study from Lockdown to Reopening. *medRxiv*. 2020;
102. Malheiro R, Figueiredo AL, Magalhães JP, et al. Effectiveness of contact tracing and quarantine on reducing COVID-19 transmission: a retrospective cohort study. *Public Health*. 2020;
103. Metlay JP, Haas JS, Soltoff AE, Armstrong KA. Household Transmission of SARS-CoV-2. *JAMA Network Open*. 2021;4(2):e210304-e210304.
104. Miyahara R, Tsuchiya N, Yasuda I, et al. Familial Clusters of Coronavirus Disease in 10 Prefectures, Japan, February− May 2020. *Emerging infectious diseases*. 2021;27(3):915.
105. Ng OT, Marimuthu K, Koh V, et al. SARS-CoV-2 seroprevalence and transmission risk factors among high-risk close contacts: a retrospective cohort study. *The Lancet Infectious Diseases*. 2021;21(3):333-343.
106. Park SY, Kim Y-M, Yi S, et al. Coronavirus Disease Outbreak in Call Center, South Korea. *Emerging Infectious Diseases*. 2020;26(8)
107. Park YJ, Choe YJ, Park O, et al. Contact Tracing during Coronavirus Disease Outbreak, South Korea, 2020. *Emerging Infectious Diseases*. October 2020;26(10)
108. Peng J, Liu J, Mann SA, et al. Estimation of secondary household attack rates for emergent spike L452R SARS-CoV-2 variants detected by genomic surveillance at a community-based testing site in San Francisco. *Clinical Infectious Diseases*. 2021;doi:10.1093/cid/ciab283
109. Pett J, McAleavey P, McGurnaghan P, et al. Epidemiology of COVID-19 in Northern Ireland, 26 February 2020–26 April 2020. *Epidemiology & Infection*. 2021;149
110. Phiriyasart F, Chantutanon S, Salaeh F, et al. Outbreak Investigation of Coronavirus Disease (COVID-19) among Islamic Missionaries in Southern Thailand, April 2020. *OSIR Journal*. 2020;13(2)
111. Reid MJA, Prado P, Brosnan H, et al. Assessing testing strategies and duration of quarantine in contact tracing for SARS-CoV-2: a retrospective study of San Francisco's COVID-19 contact tracing program, June- August, 2020. *Open Forum Infectious Diseases*. 2021;doi:10.1093/ofid/ofab171
112. Rosenberg ES, Dufort EM, Blog DS, et al. COVID-19 Testing, Epidemic Features, Hospital Outcomes, and Household Prevalence, New York State-March 2020. *Clin Infect Dis*. May 8 2020;doi:10.1093/cid/ciaa549
113. Semakula M, Niragire F, Umutoni A, et al. The secondary transmission pattern of COVID-19 based on contact tracing in Rwanda. *BMJ Global Health*. 2021;6(6):e004885. doi:10.1136/bmjgh-2020-004885

114. Seto J, Aoki Y, Komabayashi K, et al. Epidemiology of coronavirus disease 2019 in Yamagata Prefecture, Japan, January-May 2020: The importance of retrospective contact tracing. *Jpn J Infect Dis*. Mar 31 2021;doi:10.7883/yoken.JJID.2020.1073
115. Son H, Lee H, Lee M, et al. Epidemiological characteristics of and containment measures for coronavirus disease 2019 in Busan Metropolitan City, South Korea. *Epidemiol Health*. Jun 1 2020:e2020035. doi:10.4178/epih.e2020035
116. Shah K, Desai N, Saxena D, Mavalankar D, Mishra U, Patel GC. Household Secondary Attack Rate in Gandhinagar district of Gujarat state from Western India. *medRxiv*. 2020:2020.09.03.20187336. doi:10.1101/2020.09.03.20187336
117. Sun WW, Ling F, Pan JR, et al. [Epidemiological characteristics of 2019 novel coronavirus family clustering in Zhejiang Province]. *Zhonghua Yu Fang Yi Xue Za Zhi*. Mar 15 2020;54(0):E027. doi:10.3760/cma.j.cn112150-20200227-00199
118. Sunday V, Bhaskar E. Low secondary transmission rates of SARS-CoV-2 infection among contacts of construction laborers at open air environment. *Germs*. 2021;11(1):128-131.
119. Tak P, Rohilla J. COVID-19 contact tracing in a tertiary care hospital: A retrospective chart review. *Infectious Disease Modelling*. 2021;6:1-4. doi:10.1016/j.idm.2020.10.014
120. Telle K, Jorgensen SB, Hart RK, Greve-Isdahl M, Kacelnik O. Secondary attack rates of COVID-19 in Norwegian families: a nation-wide register-based study. *European Journal of Epidemiology*. 05/25 2021;doi:10.1007/s10654-021-00760-6
121. Trunfio M, Longo BM, Alladio F, et al. On the SARS-CoV-2 “Variolation Hypothesis”: No Association Between Viral Load of Index Cases and COVID-19 Severity of Secondary Cases. Hypothesis and Theory. *Frontiers in Microbiology*. 2021-March-16 2021;12(473)doi:10.3389/fmicb.2021.646679
122. Vallès X, Roure S, Valerio L, et al. SARS-CoV-2 contact tracing among disadvantaged populations during epidemic intervals should be a priority strategy: results from a pilot experiment in Barcelona. *Public Health*. 2021;
123. van der Hoek W, Backer JA, Bodewes R, et al. [The role of children in the transmission of SARS-CoV-2]. *Nederlands tijdschrift voor geneeskunde*. Jun 3 2020;164De rol van kinderen in de transmissie van SARS-CoV-2.
124. Verberk J, de Hoog M, Westerhof I, et al. Transmission of SARS-CoV-2 within households: a prospective cohort study in the Netherlands and Belgium – Interim results. *medRxiv*. 2021:2021.04.23.21255846. doi:10.1101/2021.04.23.21255846
125. Wang X, Pan Y, Zhang D, et al. Basic epidemiological parameter values from data of real-world in mega-cities: the characteristics of COVID-19 in Beijing, China. *BMC Infectious Diseases*. 2020/07/20 2020;20(1):526. doi:10.1186/s12879-020-05251-9
126. Wang Y, Tian H, Zhang L, et al. Reduction of secondary transmission of SARS-CoV-2 in households by face mask use, disinfection and social distancing: a cohort study in Beijing, China. *BMJ Global Health*. 2020;5(5):e002794.
127. Wilkinson K, Chen X, Shaw S. Secondary attack rate of COVID-19 in household contacts in the Winnipeg Health Region, Canada. *Canadian Journal of Public Health*. 2021/02/01 2021;112(1):12-16. doi:10.17269/s41997-020-00451-x
128. Wu J, Huang Y, Tu C, et al. Household Transmission of SARS-CoV-2, Zhuhai, China, 2020. *Clin Infect Dis*. May 11 2020;doi:10.1093/cid/ciaa557
129. Wu P, Liu F, Chang Z, et al. Assessing asymptomatic, pre-symptomatic and symptomatic transmission risk of SARS-CoV-2. *Clinical Infectious Diseases*. 2021;doi:10.1093/cid/ciab271
130. Wu Y, Song S, Kao Q, Kong Q, Sun Z, Wang B. Risk of SARS-CoV-2 infection among contacts of individuals with COVID-19 in Hangzhou, China. *Public Health*. 2020/08/01/ 2020;185:57-59. doi:https://doi.org/10.1016/j.puhe.2020.05.016
131. Xin H, Jiang F, Xue A, et al. Risk factors associated with occurrence of COVID-19 among household persons exposed to patients with confirmed COVID-19 in Qingdao Municipal, China. *Transboundary and emerging diseases*. Jul 20 2020;doi:10.1111/tbed.13743
132. Yung CF, Kam KQ, Chong CY, et al. Household Transmission of SARS-CoV-2 from Adults to Children. *The Journal of Pediatrics*. Jul 4 2020;doi:10.1016/j.jpeds.2020.07.009

© 2022 Madewell ZJ et al. *JAMA Network Open.*

133. Zhang JZ, Zhou P, Han DB, et al. [Investigation on a cluster epidemic of COVID-19 in a supermarket in Liaocheng, Shandong province]. *Zhonghua Liu Xing Bing Xue Za Zhi*. Apr 27 2020;41(0):E055. doi:10.3760/cma.j.cn112338-20200228-00206
134. Zhang W, Cheng W, Luo L, et al. Secondary Transmission of Coronavirus Disease from Presymptomatic Persons, China. *Emerging Infectious Diseases*. 2020;26(8)
135. Zhuang YL, Zhang YT, Li M, et al. [Analysis on the cluster epidemic of coronavirus disease 2019 in Guangdong Province]. *Zhonghua Yu Fang Yi Xue Za Zhi*. Jul 6 2020;54(7):720-725. doi:10.3760/cma.j.cn112150-20200326-00446
136. Madewell ZJ, Yang Y, Longini IM, Jr., Halloran ME, Dean NE. Factors Associated With Household Transmission of SARS-CoV-2: An Updated Systematic Review and Meta-analysis. *JAMA Netw Open*. Aug 2 2021;4(8):e2122240. doi:10.1001/jamanetworkopen.2021.22240
137. Arnedo-Pena A, Sabater-Vidal S, Meseguer-Ferrer N, et al. COVID-19 secondary attack rate and risk factors in household contacts in Castellon (Spain): Preliminary report. *Enfermedades Emergentes*. 2020;19(2):64-70.
138. Carazo S, Laliberté D, Villeneuve J, et al. Characterization and evolution of infection control practices among SARS-CoV-2 infected healthcare workers of acute care hospitals and long-term care facilities in Quebec, Canada, Spring 2020. *Infection Control & Hospital Epidemiology*. 2021:1-37. doi:10.1017/ice.2021.160
139. Patel A, Charani E, Ariyanayagam D, et al. New-onset anosmia and ageusia in adult patients diagnosed with SARS-CoV-2 infection. *Clin Microbiol Infect*. Jun 2 2020;doi:10.1016/j.cmi.2020.05.026
140. Teherani MF, Kao CM, Camacho-Gonzalez A, et al. Burden of illness in households with SARS-CoV-2 infected children. *Journal of the Pediatric Infectious Diseases Society*. Aug 11 2020;doi:10.1093/jpids/piaa097
141. Tibebu S, A. Brown K, Daneman N, Paul LA, Buchan SA. Household secondary attack rate of COVID-19 by household size and index case characteristics. *medRxiv*. 2021:2021.02.23.21252287. doi:10.1101/2021.02.23.21252287
142. Lee M, Eun Y, Park K, Heo J, Son H. Follow-up investigation of asymptomatic COVID-19 cases at diagnosis in Busan, Korea. *Epidemiology and health*. 2020;42
143. Fung HF, Martinez L, Alarid-Escudero F, et al. The household secondary attack rate of severe acute respiratory syndrome coronavirus 2 (SARS-CoV-2): a rapid review. *Clinical Infectious Diseases*. 2021;73(Supplement_2):S138-S145.

© 2022 Madewell ZJ et al. *JAMA Network Open.*
